# New Metabolites Isolated from a *Laurencia obtusa* Population Collected in Corsica

**DOI:** 10.3390/molecules23040720

**Published:** 2018-03-21

**Authors:** Hélène Esselin, Félix Tomi, Ange Bighelli, Sylvain Sutour

**Affiliations:** 1 Université de Corse—CNRS, UMR 6134 SPE, Equipe Chimie et Biomasse, Route des Sanguinaires, 20000 Ajaccio, France; helene.esselin@gmail.com (H.E.); felix.tomi@univ-corse.fr (F.T.); bighelli@univ-corse.fr (A.B.); 2 Neuchâtel Platform of Analytical Chemistry, University of Neuchâtel, Avenue de Bellevaux 51, 2000 Neuchâtel, Switzerland

**Keywords:** *Laurencia obtusa*, NMR, non terpenic, C_15_-acetogenin, *abeo*-labdane

## Abstract

The chemical investigation of an ethyl acetate extract (EtOAc) obtained from *Laurencia obtusa*, collected in Corsica, allowed for the identification of three new compounds (**1**, **2**, and **4**) and six known compounds. Compounds **1** to **4** were isolated and fully characterized by a detailed spectroscopic analysis. Compounds **1** and **2** are two C_15_-acetogenins sharing the same ring system: a tetrahydropyran linked by a methylene to a tetrahydrofuran ring. Compound **1** exhibits a bromoallene unit whereas compound **2** possesses an uncommon α-bromo-α,β-unsaturated aldehyde terminal unit. Compound **4** is the first diterpene exhibiting a 19(4→3)*abeo*-labdane skeleton isolated from a *Laurencia* species. Isolation of concinndiol (compound **3**) together with compound **4** suggests a common biosynthetic origin. Additionally, five known compounds, namely sagonenyne, laurene, α-bromocuparene, microcladallene A, and β-snyderol were identified in chromatographic fractions by NMR analysis using a computerized method that was developed in our laboratory.

## 1. Introduction

Due to its wide chemodiversity, the genus *Laurencia* has been one of the most studied genera among marine organisms. Indeed, the chemistry of *Laurencia* populations has been the subject of a number of investigations because of the large amounts of various halogenated metabolites that are produced by these populations. Besides sesquiterpenes [1] or diterpenes [2], the *Laurencia* species are known to produce C_15_ non-terpenic metabolites known as acetogenins. Most of these molecules are halogenated *O*-bridged cyclic ethers, exhibiting conjugated enyne, bromoallene, or bromopropargylic terminus [3]. They are biosynthesized from C_16_ fatty acid derivatives and may be regarded as chemotaxonomical markers of the *Laurencia* complex [3,4,5]. Diterpenes isolated from the *Laurencia* species or mollusks feeding on them (especially from the genus *Aplysia*) are divided into approximately twenty skeletons and one hundred compounds. Labdane-related compounds isolated from the *Laurencia* species are mostly brominated and represent more than half of the diterpenes [6].

*Laurencia obtusa,* the type species of the genus *Laurencia,* originates from southern England but is now widespread all over the world in warm and temperate waters, such as the Mediterranean Sea [7]. As part of our ongoing work on secondary metabolites of the *Laurencia* species, an ethyl acetate extract (EtOAc) of a *Laurencia obtusa* (Hudson) J.V. Lamouroux population, collected in Erbalunga (Corsica, France), was subjected to chromatographic fractionation in order to afford two new C_15_-acetogenins (compounds **1** and **2**) and one new rearranged labdane derivative (compound **4**). Their structures were elucidated based on a detailed interpretation of 1D and 2D NMR, and mass spectrometry. A biogenetic pathway leading to the new acetogenins **1**-**2** and diterpene **4** is also proposed.

## 2. Results

The population of *Laurencia obtusa,* collected in Erbalunga (Corsica, France), was air-dried, frozen with liquid nitrogen, reduced to powder, and then extracted at room temperature with EtOAc. The extract was analyzed using a computerized method that was developed in our laboratory based on ^13^C NMR [8]. This method allowed for the identification of individual components with limited fractionations, by comparison of the signals of the mixture spectrum with those of reference spectra present in a laboratory-built library. This revealed that the extract was dominated by a non-identified compound (**1**). Therefore, the extract was subjected to chromatographic fractionation, which led to the isolation of compounds **1** to **4** and to the identification of five known compounds: sagonenyne, laurene, α-bromocuparene, microcladallene A, and β-snyderol (Figure S29).

### Structure Elucidation of Compounds ***1**–**4***

The molecular formula of compound **1**, C_15_H_21_Br_3_O_3_, was determined by HRESIMS and from NMR data (Table 1, Figures S1–S7). Interrogation of our ^13^C NMR database suggested that there were similarities to the previously described sagonenyne (compound **5**) [9]. Interpretation of 1D and 2D NMR spectra confirmed that compound **1** shared the same 10-hydroxy-12-bromo-13-ethyltetrahydropyran ring as sagonenyne. However, the differences between the chemical shifts of both of the molecules (Δδ) were larger than 0.50 ppm for seven out of 15 carbons, indicating that half of the molecules exhibited different structural features. Furthermore, compound **1** lacked the acetoxyl group and exhibited a bromoallene unit [*δ*_H_ 6.11 (1H, dd, *J* = 5.7, 1.4 Hz, H-1) and 5.66 (1H, dd, *J* = 7.2, 5.7 Hz, H-3); *δ*_C_ 73.94 (C-1), 201.63 (C-2) and 102.01 (C-3)] instead of an en-yne unit. Besides the tetrahydropyran (THP) ring, the ^1^H and ^13^C NMR spectra of compound **1** showed resonances for three methines, one of which was linked to a bromine atom [*δ*_H_ 4.46 (1H, ddd, *J* = 6.2, 3.5, 1.8 Hz, H-6); *δ*_C_ 53.08 (C-6)], while the other two were linked to oxygen atoms [*δ*_H_ (4.64, 1H, dddd, *J* = 9.0, 7.2, 4.4, 1.4 Hz, H-4) and 3.89 (1H, ddd, *J* = 9.5, 3.5, 2.3 Hz, H-7); *δ*_C_ 74.83 (C-4) and 79.09 (C-7)]. The presence of three bromine atoms was confirmed by the typical isotopic pattern which was obtained by HRESIMS. The molecular formula of compound **1** indicated four degrees of unsaturation, revealing that compound **1** possessed a second cycle. Indeed, multiplicity and ^13^C NMR chemical shifts of C-4 and C-7 were characteristic of a tetrahydrofuran (THF) ring linked between those carbons [10]. Finally, compound **1** was identified as a C_15_-acetogenin exhibiting a bromoallene unit and a THF ring linked by a methylene to a THP ring.

**Figure 1:**
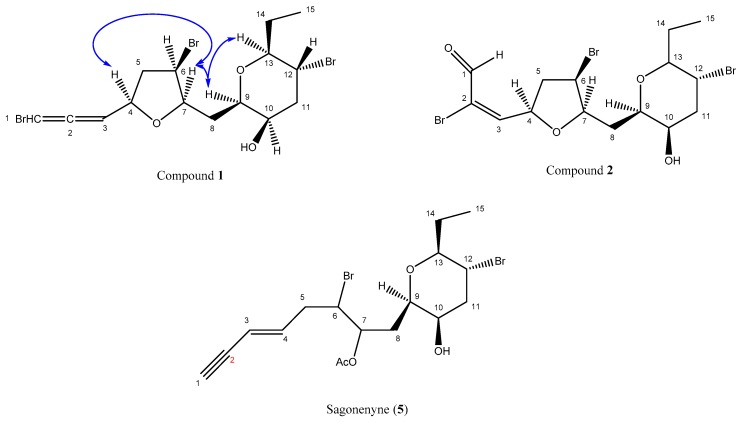
Structures of compounds **1**, **2,** and sagonenyne (**5**). Key NOESY correlations of compound **1** are represented by blue arrows.

The determination of the relative configuration of stereogenic centers of compound **1** was based on coupling constant analysis and NOESY spectra. Protons H-9, H-10, H-12, and H-13 exhibited similar coupling constant values to the previously described sagonenyne. Furthermore, the presence of a NOESY correlation between H-13 and H-9 confirmed a *syn* orientation of the THP ring linkage. Coupling constants of H-10 (^3^*J*_H10-H9_ = 1.1 Hz; ^3^*J*_H10-H11a_ = 3.2 Hz) were consistent with a *syn* orientation of the hydroxyl. Conversely, the large coupling constants between H-12 and H-13 (10.2 Hz) and between H-12 and H-11b (12.3 Hz) indicated that the bromine was *anti*-oriented compared with all other substituents of the THF ring. Moreover, a NOESY cross-peak observed between H-7 and H-4 indicated a *syn* orientation of the THF ring linkage. The small coupling constant between H-6 and H-7 (3.5 Hz) showed that the bromine also had a *syn* orientation, indicating an *R* relative configuration of C-6. In addition, a NOESY cross-peak observed between H-7 and H-9 allowed for linking the relative configuration of both THF and THP rings, as shown on Figure 1. Finally, the relative configuration of the molecule was established as 4R*,6R*,7R*,9R*,10R*,12R*,13S* (Figure 1).

The elemental composition of compound **2** was deduced as C_15_H_21_Br_3_O_4_ based on the HRESIMS and NMR data (Figures S8–S13). ^1^H and ^13^C NMR data (Table 1) of compound **2** were highly similar to those of compound **1**. 1D and 2D NMR spectra confirmed the presence of both THP and THF rings exhibiting the same substitution pattern (two bromines and one hydroxyl) as compound **1**. The most significant differences were observed for the chemical shifts of C-1 to C-3, indicating a different terminal unit for compound **2**. ^1^H, ^13^C, and HSQC NMR spectra of compound **2** revealed the presence of an α,β-unsaturated aldehyde functionalized in α position [*δ*_H_ 9.22 (1H, s, H-1) and 7.54 (1H, d, *J* = 6.3 Hz, H-3); *δ*_C_ 185.19 (C-1), 126.11 (C-2) and 156.11 (C-3)]. According to ^13^C NMR chemical shifts, multiplicity, the molecular formula, and the HRMS isotopic pattern, C-2 was bonded to a bromine.

The relative configuration of the stereogenic centers was determined based on H,H-coupling constant analysis and NOESY data. Coupling constant values of H-4, H-7, H-9, and H-13 were identical to those observed for compound **1,** indicating that both THF and THP ring linkages had a *syn* orientation. Moreover, the large coupling constant between H-12 and H-13 (10.1 Hz), as well as the small coupling constants between H-9 and H-10 (1.1 Hz), and between H-6 and H-7 (3.0 Hz), led to the same conclusions as for compound **1**: except the C-12 substitution orientation, all other substituents exhibited a *syn* relative orientation. Therefore, compound **2** exhibited the same relative configuration as **1**, namely 4R*,6R*,7R*,9R*,10R*,12R*,13S* (Figure 1).

The molecular formula of compound **3**, C_20_H_35_BrO_2_, was established by HRESIMS (Figure S20). An interrogation of our ^13^C NMR database provided no suitable candidate for this compound. However, the ^13^C chemical shifts of compound **3** recorded in acetone-*d*_6_ matched perfectly with those of concinndiol [11,12]. Since NMR data of concinndiol has remained incomplete up to this point, we report here its complete and assigned list of ^1^H and ^13^C NMR data (Table 2, Figures S14–S19) for the first time. The assignment of OH signals was based on the HMBC spectrum. Long-range correlation cross-peaks were observed between the OH resonance at 3.04 ppm and carbons C-8, C-9, C-10, and C-11, as well as between the signal at 3.71 ppm, and carbons C-12 and C-14.

The molecular formula of compound **4** was established by HRESIMS (Figures S27–S28) to be C_20_H_34_O_2,_ indicating four degrees of unsaturation. An interpretation of 1D and 2D NMR data (Table 2, Figures S21–S26 acetone-*d*_6_) showed that compound **4** exhibited one exocyclic double bond [*δ*_H_ (4.77, 1H, t, *J* = 2.1 Hz, H-18a) and 4.41 (1H, t, *J* = 2.1 Hz, H-18b); *δ*_C_ 157.43 (C-4) and 106.62 (C-18)] and a terminal double bound in α-position of an alcohol function [*δ*_H_ (5.92, 1H, dd, *J* = 17.3, 10.7 Hz, H-14), 5.21 (1H, dd, *J* = 17.3, 1.9 Hz, H-15a) and 4.97 (1H, dd, *J* = 10.7, 1.9 Hz, H-15b); *δ*_C_ 73.32 (C-13), 147.15 (C-14) and 111.34 (C-15)]. Additionally, compound **4** showed resonances of four methyls, six methylenes, three methines, and two quaternary carbons, one of which was oxygenated [*δ*_C_ 76.51 (C-9)]. All of these data matched with a labdane structure similar to the one of compound **3**. The largest chemical shift differences (Δδ >2.00 ppm) between compounds **4** and **3** were observed on C-1 to C-5, indicating that the modification between both compounds occurred on cycle A. Indeed, 2D NMR data showed that the bromine atom bore by C-3 in compound **3** was substituted by a methyl. This observation is consistent with the molecular formula deduced from HRMS and the shielded chemical shift of C-3 compared with compound **3** (39.53 vs. 71.35). Moreover, HMBC spectra allowed for locating the exocyclic double bond on C-4, adding a second modification on cycle A. Finally, the structure of compound **4** was deduced to be a 19(4 → 3)*abeo*-labdane. This kind of skeleton had been previously isolated from the Mediterranean sponge *Mycale rotalis* [13]. Indeed, rotalin A is the (9-13)-epoxy analogue of compound **4**. ^13^C chemical shifts of both molecules were compared and no significant difference was observed (Δ*δ* < 1.35 ppm).

**Figure 2:**
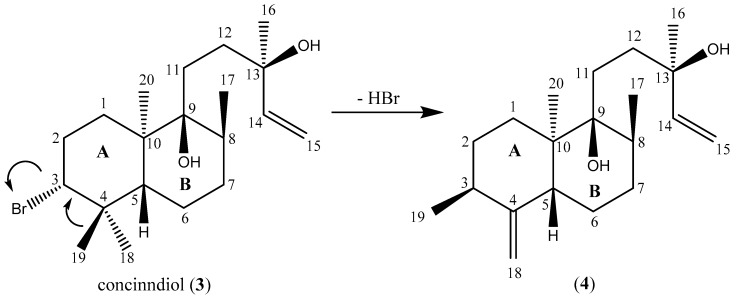
Biosynthetic pathway from concinndiol (compound **3**) to compound **4**.

The relative configuration of C-3, C-5, C-8, and C-10 was determined based on NOESY data. NOESY cross-peaks observed from H-19 to H-1a, H-2b, and H-5, along with correlations from H-5 to H-1a and H-7a, as well as from H-17 to H-7a, indicated that H-5 and methyls C-17 and C-19 had a *syn* orientation. NOESY cross-peaks observed from H-20 to H-1b and H-8 showed that methyl C-20 was positioned on the other side of the molecule. Concerning C-9 and C-13, the relative configuration was established by a comparison of chemical shifts with the data contained in the literatures. Both carbons exhibited a Δ*δ* <0.40 ppm with concinndiol or rotalin A, indicating the same relative configuration. Therefore, compounds **3** and **4** share the same relative configuration, except for carbon C-3. This observation is consistent with the hypothetic pathway from concinndiol to compound **4** inspired by the biosynthetic formation of pannosanol [6] (Figure 2).

## 3. Discussion

The group of acetogenins containing six-membered cyclic ethers is one of the smallest groups among *Laurencia* acetogenins. It includes 14 compounds which can be divided into four subgroups: those containing only one tetrahydropyran ring, those containing both tetrahydropyran and tetrahydrofuran rings (fused or not), and those containing two fused tetrahydropyran rings [14]. To the best of our knowledge, only two natural C_15_-acetogenins containing separated tetrahydropyran and tetrahydrofuran rings were previously described [4]. One had been isolated from a *Laurencia obtusa* specimen from the Canary Islands [10] and another from the sponge *Mycale Rotalis* [15], and later from a *Laurencia paniculata* specimen from Turkey [16] and from a *Laurencia obtusa* population that was collected in Greece [17]. They both bear different substitution patterns from compounds **1** and **2** and are characterized by a bromopropargylic terminal unit. Furthermore, only one C_15_-acetogenin containing a halogenated aldehyde terminus has been described [18]. This linear metabolite was isolated from *L. nidifica* [19] and is supposed to be synthesized from its corresponding enyne hydrocarbon.

**Figure 3:**
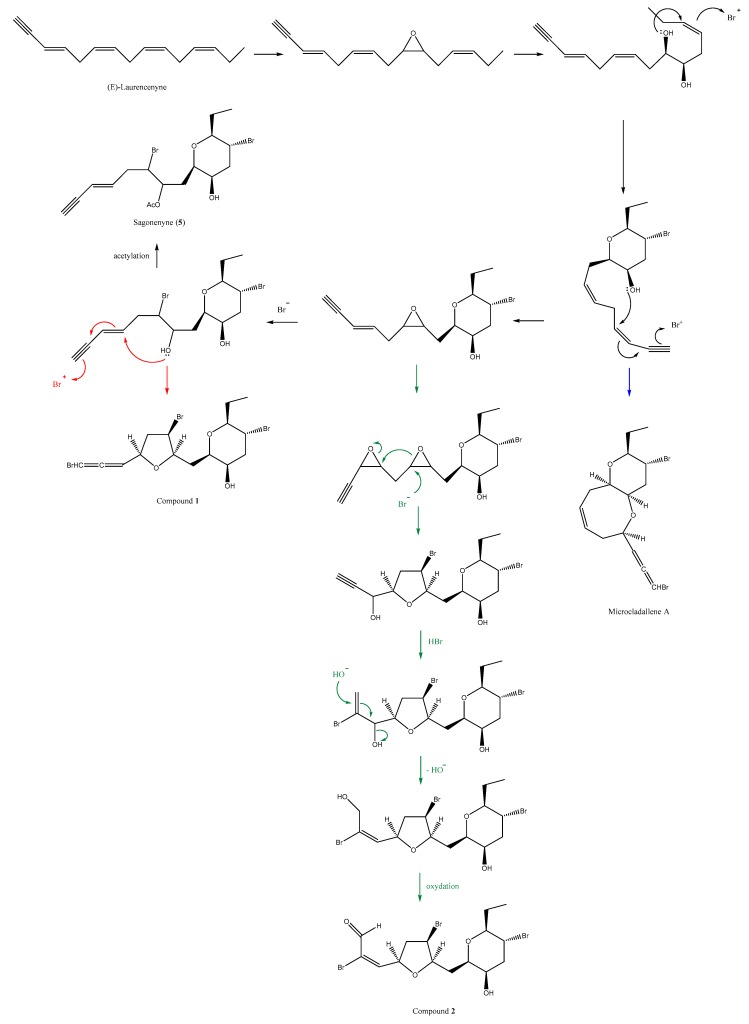
Hypothetic biogenesis of compounds **1**, **2,** and microcladallene A.

Compounds **1**, **2**, and sagonenyne (**5**) are the only examples of acetogenins containing the same (9R*,10R*,12R*,13S*)-10-hydroxy-12-bromo-13-ethyltetrahydropyran ring (Scanlonyne exhibiting a 9,13-*trans* THP ring [20]). Consequently, we suggest a common biogenesis (Figure 3) of those compounds starting from *trans*-laurencenyne, a C_15_ acetylenic polyene isolated from a *L. okamurai* population [21]. Indeed, a first (9,10)-epoxydation followed by a (9,13)-cyclization through the oxygen atom allows the formation of a common precursor for compounds **1**, **2**, and sagonenyne (**5**). Additionally, microcladallene A (compound **8**), sharing the same relative configuration of the THP ring, could also be obtained from this compound. A second (6,7)-epoxydation, followed by a C-6 bromination may lead to sagonenyne (compound **5**) after acetylation or to compound **1** after (4,7)-THF ring closure. As shown in Figure 3, a series of hypothetic rearrangements and oxidation from the (6,7)-epoxy derivative may generate compound **2**. The fact that these compounds likely all share a common origin suggests that they may constitute interesting taxonomical markers of the *Laurencia obtusa* species from Corsica.

Compared to C_15_-acetogenins or sesquiterpenes, diterpenes represent a small group among *Laurencia* secondary metabolites. Labdanes and related metabolites are the largest group of diterpenes isolated from the *Laurencia* species or mollusks grazing on them, and account for about 50% of the *Laurencia* diterpenes [2]. Among them, only two labdane derivatives bear a hydroxyl on C-9 (concinndiol and neoconncindiol hydroperoxyde) and all of them exhibit a bromine substitution on C-3, except laukarlaol and neoconcinndiol hydroperoxyde [14]. Altogether, the 19(4→3)*abeo*-labdane skeleton isolated in this study has never been reported in *Laurencia,* and thus represents a highly original structure to be added to the already extensive chemical diversity of this genus.

## 4. Materials and Methods

### 4.1. General Experimental Procedures

The NMR Spectra were recorded on a Bruker AVANCE 400 Fourier Transform spectrometer (Bruker, Wissembourg, France) operating at 400.13 MHz for ^1^H and at 100.13 MHz for ^13^C, using CDCl_3_ or acetone-*d*_6_ with TMS (Tetramethylsilane) as the internal standard. COSY spectra were recorded using 512 data points in F2 dimension and 256 data points in F1 dimension, a spectral width (SW) of 12 ppm in both dimensions, and an accumulation of 32 scans. HSQC and HMBC spectra were recorded using 512 data points in F2 and 256 data points in F1 dimension, SW of 12 ppm in F2 dimension (^1^H) and of 220 ppm in F1 dimension (^13^C), and an accumulation of 64 scans. NOESY spectra were recorded using the Bruker microprogram (PULPROG noesyph, Bruker, Wissembourg, France), a mixing time of 300 ms, 1024 data points in F2 dimension and 256 data points in F1 dimension, a SW of 12 ppm in both dimensions, and an accumulation of 64 scans.

High resolution mass spectra were obtained by injecting samples dissolved in MeOH on a Synapt G2 QTOF mass spectrometer (Waters, Milford, MA, USA) in both electrospray positive and negative modes. The following conditions were employed: capillary voltage, 2.8 kV (positive mode) and −2.0 kV (negative mode); source block temperature, 120 °C; desolvation gas (nitrogen) flow and temperature 400 °C and 800 L/h; cone voltage +25 V (positive mode) and −25 V (negative mode). The mass range was 85–1200 Da, MS scan time was set to 0.4 s, and a resolution of 20,000 (FWHM at *m*/*z* 500) was selected. Sub-2 ppm mass accuracy was obtained by infusing a 0.5 mM solution of sodium formate, as an external calibrant prior to measurements, and a 500 ng/mL solution of leucine-enkephalin, as an internal calibrant (LocksprayTM) during measurements. Data acquisition and processing were performed in Masslynx 4.1 (Waters, Milford, MA, USA).

A semi-preparative reversed phase HPLC was used to purify components (XTerra^®^ Prep MS C_18_ OBD™, Waters, Milford, MA, USA 5 µm, 19 × 150 mm) and column chromatography was performed with silica gel.

### 4.2. Plant Material

Specimens of *Laurencia obtusa* were collected at a depth of 10–20cm in June 2016, in Erbalunga (41°44′28.4″ N, 9°27′41.9″ E). The voucher specimen (H8318) was deposited at the Verlaque Herbarium, HCOM (Herbarium of Centre d'Océanologie de Marseille), Aix-Marseille Université, Institut Méditerranéen d’Océanologie, France. Identification based on molecular phylogenies was then performed by Line Le Gall (Institut de Systématique, Evolution et Biodiversité, Museum National d’Histoire Naturelle, Paris).

### 4.3. Extraction and Isolation

Algae were washed with tap water and dried with a lyophilizer. After removing the solvent under reduced pressure, the air-dried sample of *Laurencia obtusa* (152.2 g) was extracted with EtOAc at room temperature to afford a dark green crude extract (1.1305 g). This extract was subjected to silica gel column chromatography (Sigma, St. Louis, MO, USA, SiO_2_, 63–200 µm, 75 g) and eluted with a gradient of pentane/CHCl_3_/EtOAc/MeOH to afford seven fractions (F1–F7). F2, F3, and F4 (602.0 mg, 50%Pentane/50%CHCl_3_) were combined and submitted to repeated column chromatographies (SiO_2_, 35–70 µm, 30 g) using the same gradient to isolate compounds **1** to **4**. The final steps of purification were done for each compound, with the exception of compound **2**, on reversed phase semi-preparative HPLC (Waters, Milford, MA, USA) to afford compound **1** (ACN/H_2_O 0.5:0.5 to 0:1, *v*/*v*, flow rate 8 mL/min, t_R_ = 21 min), compound **3** (ACN/H_2_O 0.6:0.4 to 0:1, *v*/*v*, flow rate 8 mL/min, t_R_ = 25 min) and compound **4** (ACN/H_2_O 0.65:0.35 to 0:1, *v*/*v*, flow rate 8 mL/min, t_R_ = 17 min).

#### 4.3.1. Compound **1**

HRESIMS (Figure S7) *m*/*z* 530.9025, 532.8994, 534.8971, 536.8964 [M + HCOO]^−^ (32:100:95:34) (calcd for C_16_H_22_^79^Br_3_O_5_, 530.9018; C_16_H_22_^79^Br_2_^81^BrO_5_, 532.8997; C_16_H_22_^79^Br^81^Br_2_O_5_, 534.8977; C_16_H_22_^81^Br_3_O_5_, 536.8956); *m*/*z* 484.8986, 486.8971, 488.8927, 490.8894 [M − H]^−^ (4:10:11:4) (calcd for C_15_H_20_^79^Br_3_O_3_, 484.8963; C_15_H_20_^79^Br_2_^81^BrO_3_, 486.8942; C_15_H_20_^79^Br^81^Br_2_O_3_, 488.8622; C_15_H_20_^81^Br_3_O_3_, 490.8901); ^1^H NMR and ^13^C NMR, see Table 1.

#### 4.3.2. Compound **2**

HRESIMS (Figure S13) *m*/*z* 546.8997, 548.8940, 550.8935, 552.8925 [M + HCOO]^−^ (20:50:58:18) (calcd for C_16_H_22_^79^Br_3_O_6_, 546.8967; C_16_H_22_^79^Br_2_^81^BrO_6_, 548.8946; C_16_H_22_^79^Br^81^Br_2_O_6_, 550.8926; C_16_H_22_^81^Br_3_O_6_, 552.8905); *m*/*z* 500.8911, 502.8882, 504.8881, 506.8855 [M − H]^−^ (30:98:100:28) (calcd for C_15_H_20_^79^Br_3_O_4_, 500.8912; C_15_H_20_^79^Br_2_^81^BrO_4_, 502.8891; C_15_H_20_^79^Br^81^Br_2_O_4_, 504.8871; C_15_H_20_^81^Br_3_O_4_, 506.8851); ^1^H NMR and ^13^C NMR, see Table 1.

#### 4.3.3. Concinndiol (Compound **3**)

HRESIMS (Figure S20) *m*/*z* 351.1681, 353.1664 [M−2OH−H]^+^ (98:100) (calcd for C_20_H_32_^79^Br, 351.1687; C_20_H_32_^81^Br, 353.1667); ^1^H NMR (CDCl_3_, 400MHz) *δ* 5.91 (1H, dd, *J* = 17.3, 10.7, H-14), 5.23 (1H, dd, *J* = 17.3, 1.1, H-15a), 5.09 (1H, dd, *J* = 10.7, 1.1, H-15b), 4.05 (1H, dd, *J* = 12.0, 4.8, H-3), 2.15 (2H, m, H-2), 1.76 (1H, m, H-1a), 1.69 (1H, m, H-5), 1.67 (1H, m, H-11a), 1.63 (1H, m, H-6a), 1.63 (1H, m, H-12), 1.50 (1H, m, H-11b), 1.48 (1H, m, H-7a), 1.47 (1H, m, H-1a), 1.41 (1H, m, H-6b), 1.30 (3H, s, H-16), 1.09 (3H, s, H-19), 0.98 (3H, s, H-18), 0.98 (3H, s, H-20), 0.86 (3H, d, *J* = 6.6, H-19); ^13^C NMR (CDCl_3_, 400 MHz) *δ* 145.15 (CH, C-14), 111.92 (CH_2_, C-15), 76.52 (C, C-9), 73.53 (C, C-13), 69.93 (CH, C-3), 47.06 (C, C-5), 43.48 (C, C-10), 39.65 (C, C-4), 37.05 (CH, C-12), 36.52 (CH, C-8), 33.83 (CH_2_, C-1), 31.41 (CH_2_, C-7), 31.01 (CH_2_, C-2), 30.90 (CH_3_, C-19), 27.96 (CH_2_, C-11), 27.93 (CH_3_, C-16), 23.18 (CH_2_, C-6), 18.40 (CH_3_, C-18), 16.43 (CH_3_, C-17), 16.12 (CH_3_,C-20).

#### 4.3.4. Compound **4**

HRESIMS (Figures S27 and S28) *m*/*z* 271.2427 [M−2OH−H]^+^ (calcd for C_20_H_31_, 271.2425); *m*/*z* 351.2537 [M + HCOO]^−^ (calcd for C_21_H_35_O_4_, 351.2535); ^1^H NMR (CDCl_3_, 400MHz) *δ* 5.90 (1H, dd, *J* = 17.3, 10.7, H-14), 5.23 (1H, dd, *J* = 17.3, 1.2, H-15a), 5.07 (1H, dd, *J* = 10.7, 1.2, H-15b), 4.80 (1H, t, *J* = 1.8, H-8a), 4.44 (1H, t, *J* = 2.0), 2.52 (1H, br t, H-5), 2.50 (1H, br t, H-3), 2.02 (1H, td, *J* = 13.4, 4.3, H-1a), 1.83 (1H, m, H-8), 1.75 (1H, m, H-2a), 1.64 (1H, m, H-12), 1.62 (2H, m, H-11), 1.48 (1H, m, H-7a), 1.44 (1H, m, H-2b), 1.41 (2H, m, H-6), 1.29 (3H, s, C-16), 1.28 (1H, s, H-1b), 1.10 (3H, d, *J* = 7.2, H-19), 0.90 (3H, d, *J* = 6.6, H-17), 0.77 (3H, s, H-20); ^13^C NMR (CDCl_3_, 400 MHz) *δ* 156.06 (C, C-4), 145.24 (CH, C-14), 111.84 (CH_2_, C-15), 106.43 (CH_2_, C-18), 76.42 (C, C-9), 73.48 (C, C-13), 45.17 (C, C-10), 38.77 (CH, C-5), 38.46 (CH, C-3), 37.24 (CH, C-12), 35.62 (CH, C-8), 30.42 (CH_2_, C-7), 28.74 (CH_2_, C-11*), 28.72 (CH_2_, C-2*), 27.84 (CH_3_, C-16), 26.54 (CH_2_, C-1), 24.28 (CH_2_, C-6), 19.77 (CH_3_, C-19), 16.45 CH_3_, C-17), 14.88 (CH_3_, C-20).

## Figures and Tables

**Table 1:** NMR spectroscopic data (400 MHz, CDCl_3_) of compounds **1** and **2**.

	1		2	
	*δ*_H_ (*J* in Hz)	*δ*_C_	*δ*_H_ (*J* in Hz)	*δ*_C_
1	6.11 (dd 5.7, 1.4)	73.94	9.22 (s)	185.19
2	-	201.63	-	126.11
3	5.66 (dd 7.2, 5.7)	102.01	7.54 (d 6.3)	156.11
4	4.64 (dddd 9.0, 7.2, 4.4, 1.4)	74.83	5.09 (ddd 10.1, 6.3, 3.8)	76.56
5a	2.97 (ddd 15.0, 9.0, 6.2)	43.01	3.24 (ddd 15.0, 10.1, 5.6)	44.12
5b	2.46 (ddd 15.0, 4.4, 1.8)		2.47 (ddd 15.0, 3.8, 1.1)	
6	4.46 (ddd 6.2, 3.5, 1.8)	53.08	4.54 (ddd 5.6, 3.0, 1.1)	53.68
7	3.89 (ddd 9.5, 3.5, 2.3)	79.09	3.97 (ddd 9.2, 3.0, 2.8)	79.93
8a	2.01 (m)	36.20	2.05 (m)	36.24
8b	1.85 (ddd 14.5, 9.5, 4.1)		1.89 (ddd 14.7, 9.2, 3.7)	
9	3.71 (ddd 9.6, 4.1, 1.1)	77.16	3.71 (ddd 10.0, 3.7, 1.1)	77.22
10	3.75 (ddd 3.2, 2.8, 1.1)	69.75	3.75 (ddd 3.2, 3.0, 1.1)	69.82
11a	2.60 (ddd 13.6, 4.6, 3.2)	43.17	2.61 (ddd 13.8, 4.6, 3.2)	43.23
11b	2.13 (ddd 13.6, 12.3, 2.8)		2.14 (m)	
12	4.03 (ddd 12.3, 10.2, 4.6)	47.95	4.03 (ddd 12.4, 10.1, 4.6)	47.58
13	3.38 (ddd 10.2, 8.8, 2.3)	83.53	3.39 (ddd 10.1, 8.7, 2.4)	83.63
14a	2.06 (m)	26.36	2.07 (m)	26.32
14b	1.51 (m)		1.52 (m)	
15	0.97 (t 7.4)	9.57	0.99 (t 7.4)	9.58

**Table 2:** NMR spectroscopic data (400 MHz, acetone-*d*_6_) of compounds **3** and **4**.

	3		4	
	*δ*_H_ (*J* in Hz)	*δ*_C_	*δ*_H_ (*J* in Hz)	*δ*_C_
1a	1.88 (m)	34.55	2.14 (m)	27.21
1b	1.47 (m)		1.30 (m)	
2	2.18 (m)	32.00	1.75 (m)	29.65
2b	2.05 (m)		1.43 (m)	
3	4.07 (dd 12.7, 4.2)	71.35	2.47 (m)	39.53
4	-	40.27	-	157.43
5	1.85 (m)	47.54	2.69 (m)	39.32
6a	1.59 (m)	24.08	1.37 (m)	25.30
6b	1.44 (m)			
7a	1.42 m)	32.18	1.52 (m)	31.09
7b			1.40 (m)	
8	1.72 (m)	37.12	1.83 (m)	36.00
9	-	76.87	-	76.51
10	-	44.33	-	45.97
11a	1.67 (m)	29.20	1.66 (m)	29.85
11b	1.58 (m)			
12	1.62 (m)	38.47	1.64 (m)	38.47
13	-	73.36	-	73.32
14	5.91 (dd 17.3, 10.7)	147.17	5.92 (dd 17.3, 10.7)	147.15
15a	5.20 (dd 17.3, 1.9)	111.35	5.21 (dd 17.3, 1.9)	111.34
15b	4.96 (dd 10.7, 1.9)		4.97 (dd 10.7, 1.9)	
16	1.22 (s)	28.31	1.23 (s)	28.35
17	0.86 (d 6.6)	16.81	0.90 (d 6.6)	16.83
18a	0.96 (s)	18.74	4.77 (t 2.1)	106.62
18b			4.41 (t 2.1)	
19	1.05 (s)	31.29	1.08 (d 7.2)	20.08
20	0.99 (s)	16.65	0.78 (s)	15.49
OH (C-9)	3.04 (br s)	-	3.00 (br s)	-
OH (C-13)	3.71 (br s)	-	3.64 (br s)	-

## Supplementary Materials

The following are available online. Figure S1–S6: 1D and 2D NMR spectra of **1** in CDCl_3_, Figure S7: HRMS spectrum of **1** (Zoom) in negative ionization, Figure S8–S12: 1D and 2D NMR spectra of **2** in CDCl_3_, Figure S13: HRMS spectrum of **2** (Zoom) in negative ionization, Figure S14–S19: 1D and 2D NMR spectra of **3** in acetone-*d*_6_, Figure S20: HRMS spectrum of **3** (Zoom) in negative ionization, Figure S21–S26: 1D and 2D NMR spectra of **4** in acetone-*d*_6_, Figure S27: HRMS spectrum of **4** (Zoom) in positive ionization, Figure S28: HRMS spectrum of **4** (Zoom) in positive ionization, Figure S29: Structures of known compounds identified in *L. obtuse* extract.
