# Supplementary material for: New Metabolites Isolated from a Laurencia obtusa Population Collected in Corsica
Source: Molecules. 2018 Mar 21;23(4):720. doi: 10.3390/molecules23040720 (PMC6017553; doi:10.3390/molecules23040720)
Supplement: Supplementary file 1 [file molecules-23-00720-s001.pdf]

→SUPPLEMENTARY MATERIAL

New metabolites isolated from a *Laurencia obtusa* population collected in Corsica

Hélène Esselin [1], Félix Tomi [1], Ange Bighelli[1], and Sylvain Sutour [1,2,*]

[1]Université de Corse – CNRS, UMR 6134 SPE, Equipe Chimie et Biomasse, Route des Sanguinaires, 20000 Ajaccio, France ; helene.esselin@gmail.com (H.E.) ; felix.tomi@univ-corse.fr (F.T.) ; bighelli@univ-corse.fr (AB)
[2]Neuchâtel Platform of Analytical Chemistry, University of Neuchâtel, Avenue de Bellevaux 51, 2000 Neuchâtel, Switzerland ; sylvain.sutour@unine.ch (S.S.)
*Correspondence: sylvain.sutour@unine.ch ; Tel.: +41 (0) 32 718 24 35

Received: date; Accepted: date; Published: date

Abstract: Chemical investigation of a *Laurencia obtusa* EtOAc extract collected in Corsica allowed the identification of 3 new compounds (**1**, **2** and **4**) and 6 known compounds. Compounds **1** to **4** were isolated and fully characterized by detailed spectroscopic analysis. Compounds **1** and **2** are two C_{15}-acetogenins sharing the same ring system: a tetrahydropyran linked by a methylene to a tetrahydrofuran ring. Compound **1** exhibits a bromoallene unit whereas compound **2** possesses an uncommon α-bromo-α,β-unsatured aldehyde terminal unit. Compound **4** is the first diterpene exhibiting a 19(4→3)*abeo*-labdane skeleton isolated from a *Laurencia* species. Isolation of concinndiol (**3**) together with compound **4** suggests a common biosynthetic origin. Additionally, 5 known compounds were identified in chromatography fractions by NMR analysis following a computerized method developed in our laboratory: sagonenyne, laurene, a-bromocuparene, microcladallene A and β-snyderol.

Keywords: *Laurencia obtusa* ; NMR ; non terpenic ; C_{15} -acetogenin ; *abeo*-labdane

Figure S1: ^{1}H NMR spectrum of **1** in $CDCl_3$ (400 MHz).
Figure S2: ^{13}C NMR spectrum of **1** in $CDCl_3$ (100 MHz).
Figure S3: HSQC spectrum of **1** in $CDCl_3$.
Figure S4: HMBC spectrum of **1** in $CDCl_3$.
Figure S5: ^{1}H-^{1}H COSY spectrum of **1** in $CDCl_3$.
Figure S6: NOESY spectrum of **1** in $CDCl_3$.
Figure S7: HRMS spectrum of **1** (Zoom) in negative ionization.
Figure S8 ^{1}H NMR spectrum of **2** in $CDCl_3$ (400 MHz).
Figure S9: ^{13}C NMR spectrum of **2** in $CDCl_3$ (100 MHz).
Figure S10: HSQC spectrum of **2** in $CDCl_3$.
Figure S11: HMBC spectrum of **2** in $CDCl_3$.
Figure S12: ^{1}H-^{1}H COSY spectrum of **2** in $CDCl_3$.
Figure S13: HRMS spectrum of **2** (Zoom) in negative ionization.
Figure S14: ^{1}H NMR spectrum of **3** in acetone-d_6 (400 MHz).
Figure S15: ^{13}C NMR spectrum of **3** in acetone-d_6 (100 MHz).
Figure S16: HSQC spectrum of **3** in acetone-d_6.
Figure S17: HMBC spectrum of **3** in acetone-d_6.
Figure S18: ^{1}H-^{1}H COSY spectrum of **3** in acetone-d_6.
Figure S19: NOESY spectrum of **3** in acetone-d_6.
Figure S20: HRMS spectrum of **3** (Zoom) in negative ionization.
Figure S21: ^{1}H NMR spectrum of **4** in acetone-d_6 (400 MHz).
Figure S22: ^{13}C NMR spectrum of **4** in acetone-d_6 (100 MHz).
Figure S23: HSQC spectrum of **4** in acetone-d_6.
Figure S24: HMBC spectrum of **4** in acetone-d_6.
Figure S25: ^{1}H-^{1}H COSY spectrum of **4** in acetone-d_6.
Figure S26: NOESY spectrum of **4** in acetone-d_6.
Figure S27: HRMS spectrum of **4** (Zoom) in positive ionization.
Figure S28: HRMS spectrum of **4** (Zoom) in positive ionization.
Figure S29**.** Structures of known compounds identified in *L. obtusa* extract

Figure S1. ^{1}H NMR spectrum of compound **1** ($CDCl_3$).

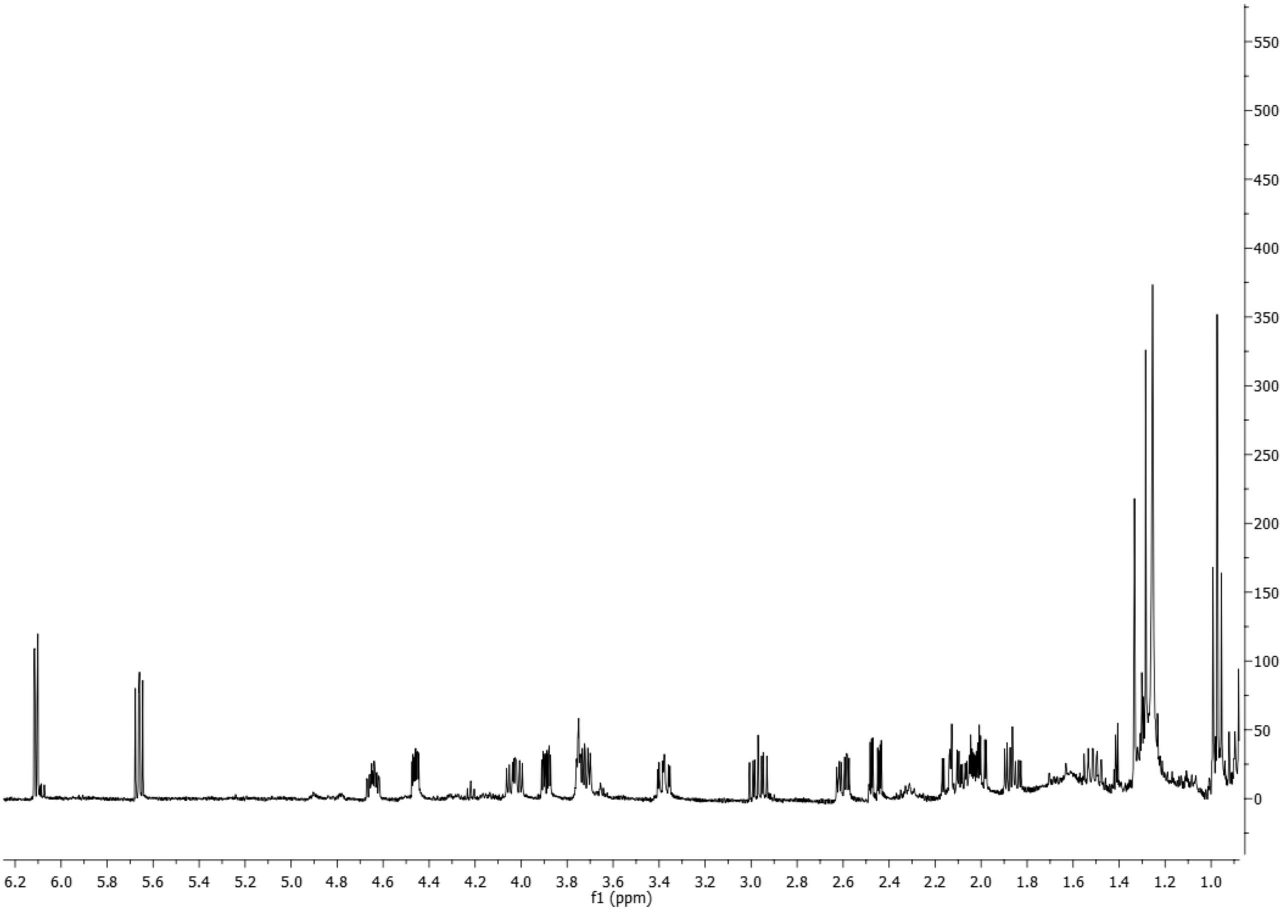

Figure S2. ^{13}C NMR spectrum of compound **1** ($CDCl_3$).

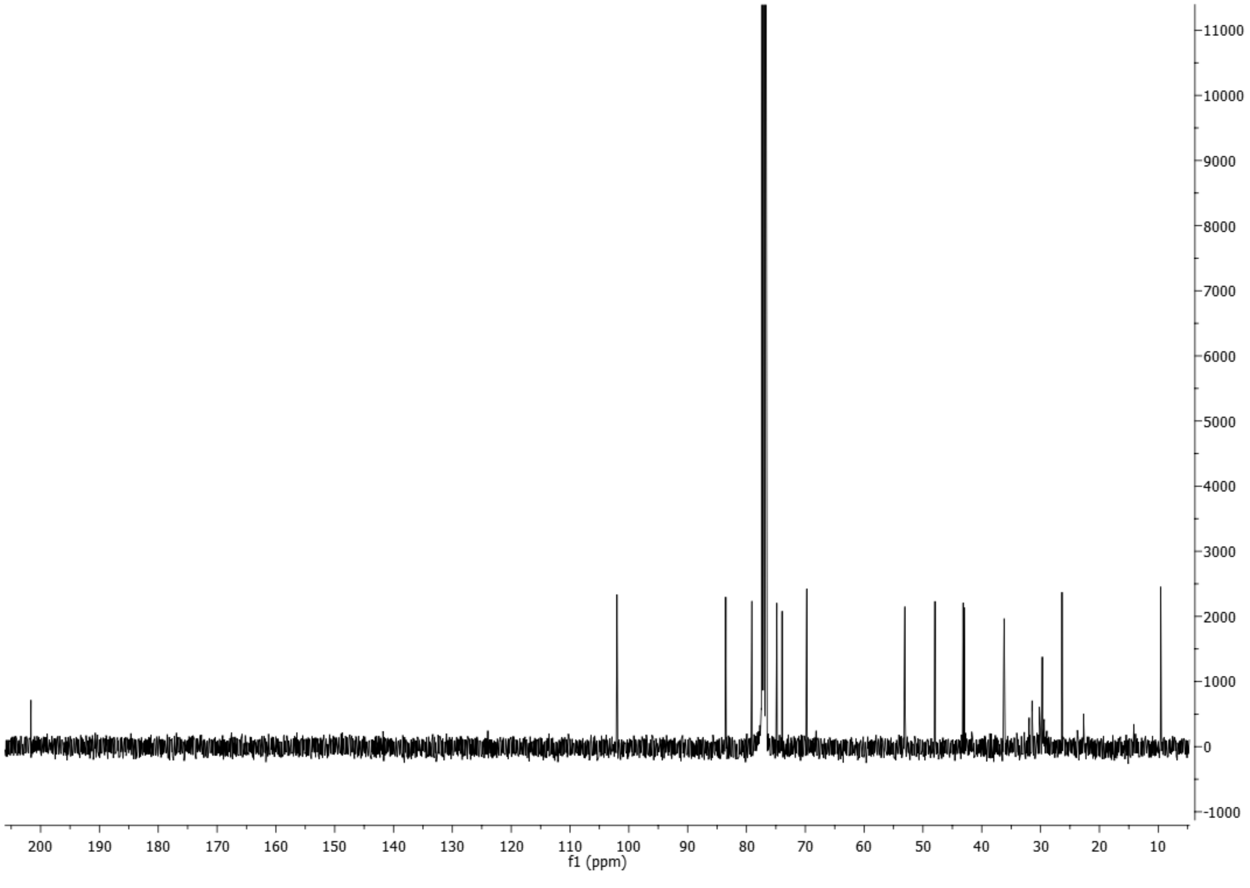

Figure S3. HSQC spectrum of compound **1** ($CDCl_3$).

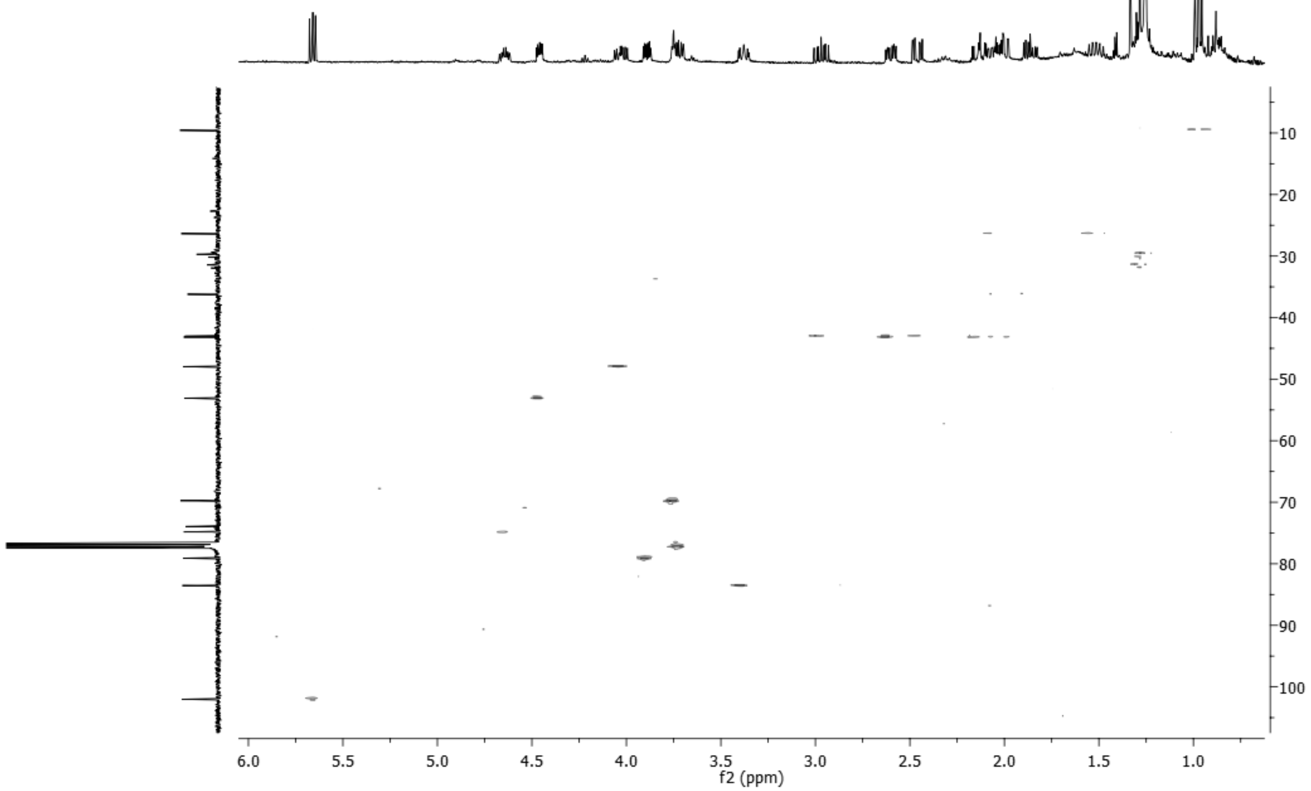

Figure S4. HMBC spectrum of compound **1** ($CDCl_3$).

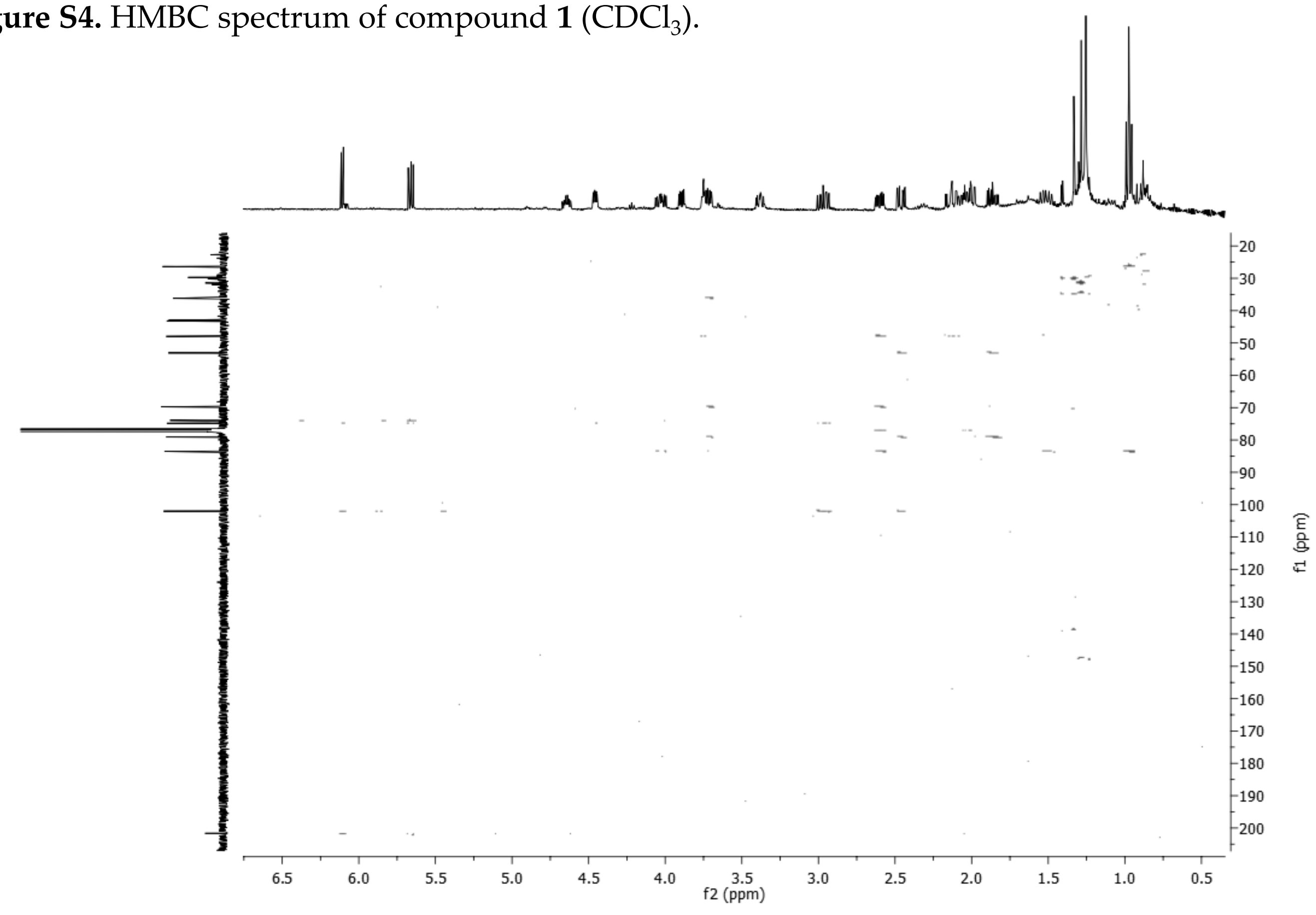

Figure S5. COSY spectrum of compound **1** ($CDCl_3$).

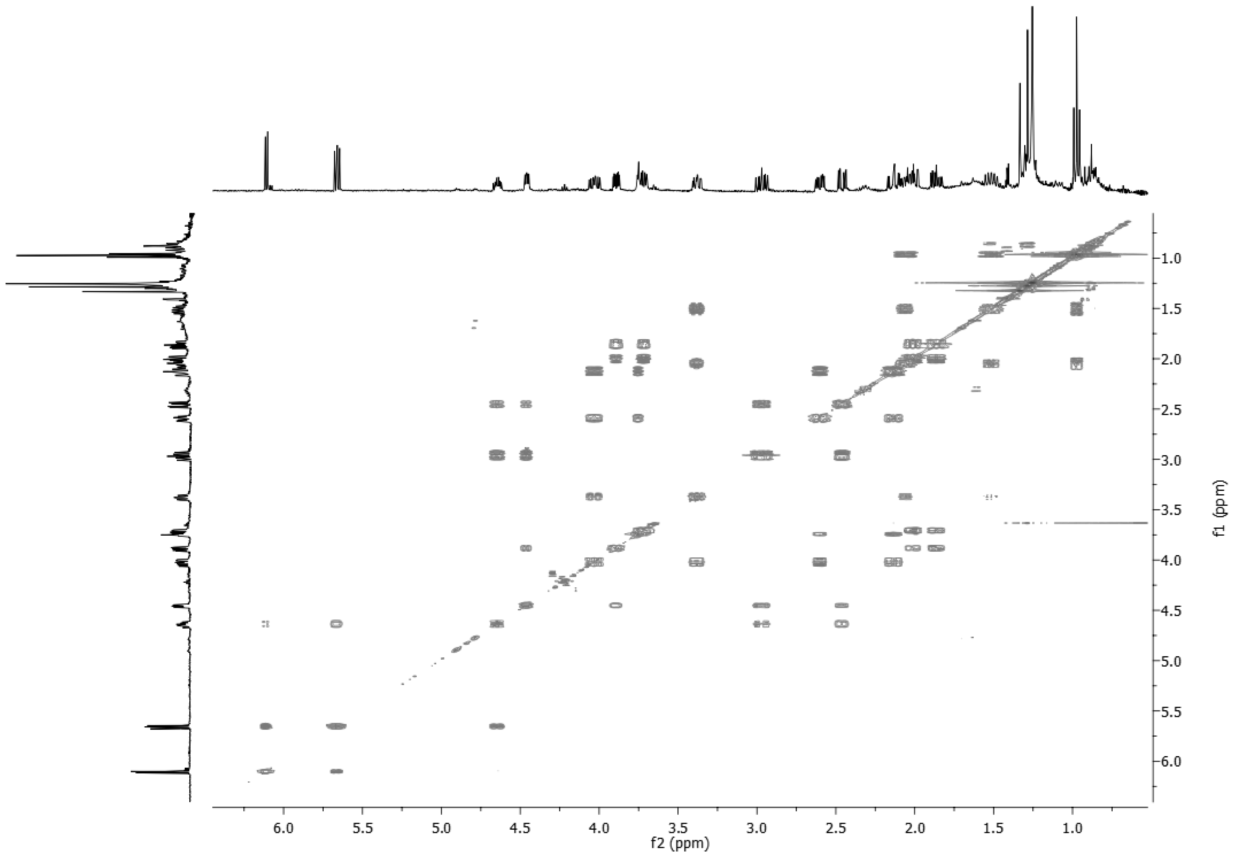

Figure S6. NOESY spectrum of compound **1** ($CDCl_3$).

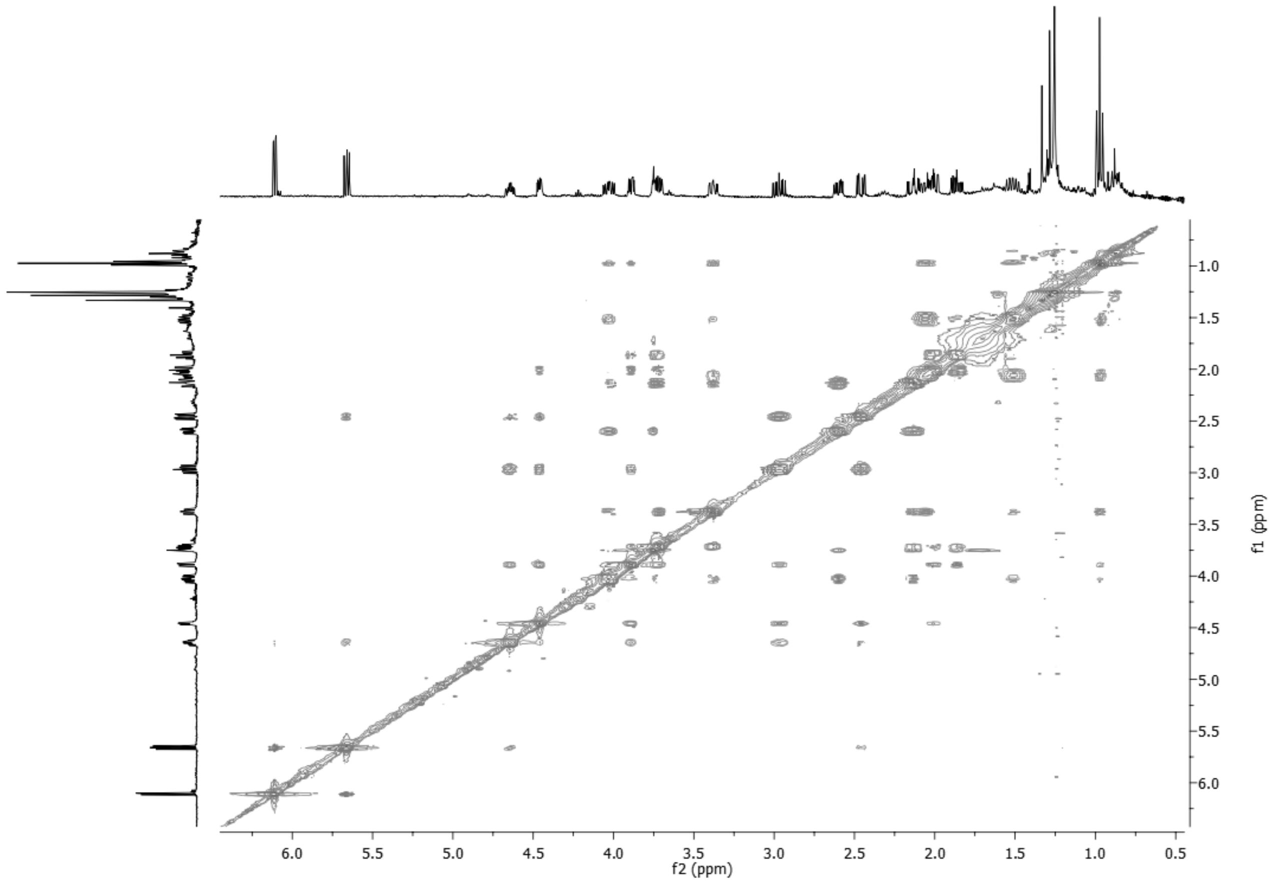

Figure S7. HRMS spectrum of compound **1** (Zoom) in negative ionization.

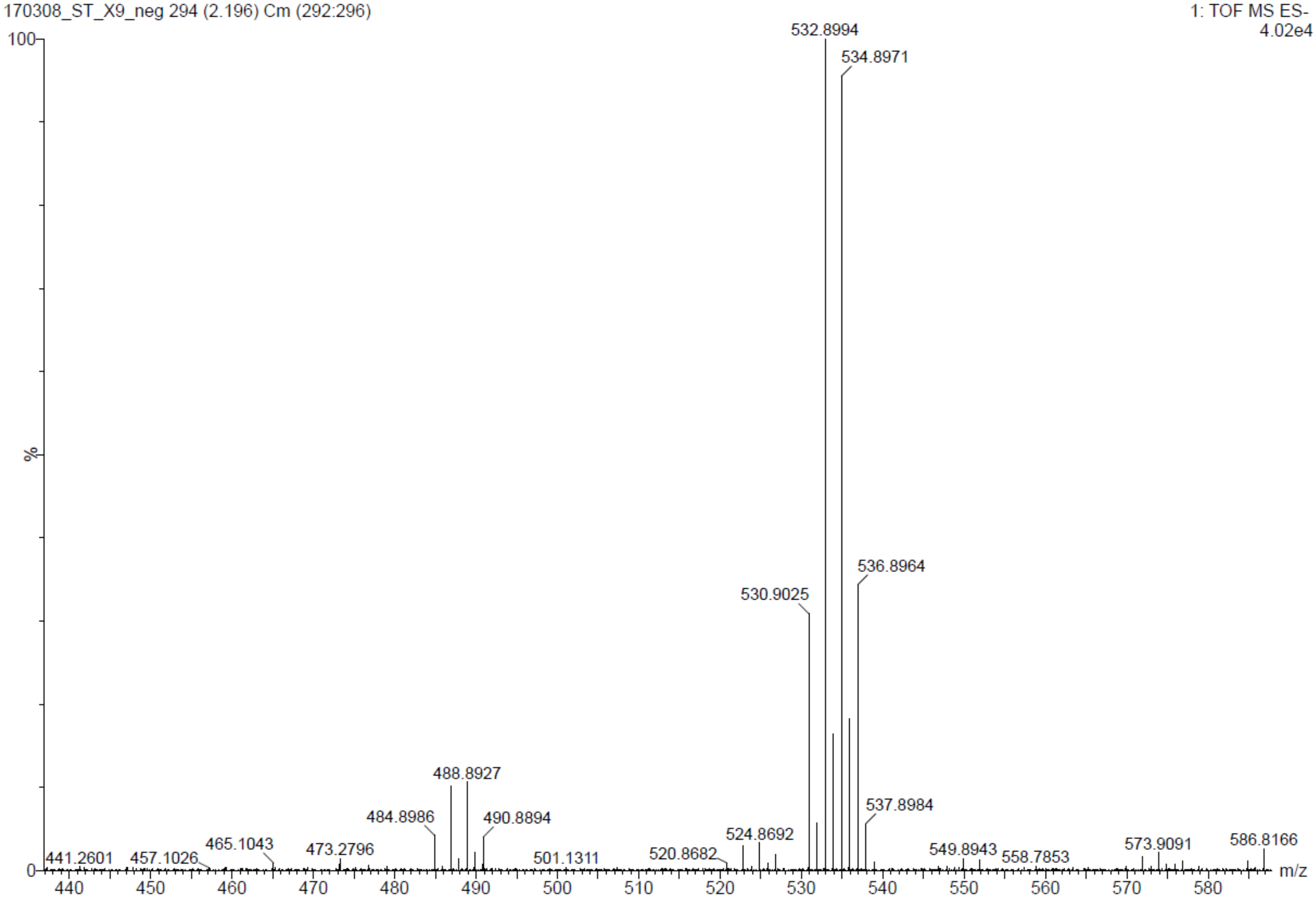

Figure S8. ^{1}H NMR spectrum of compound **2** ($CDCl_3$).

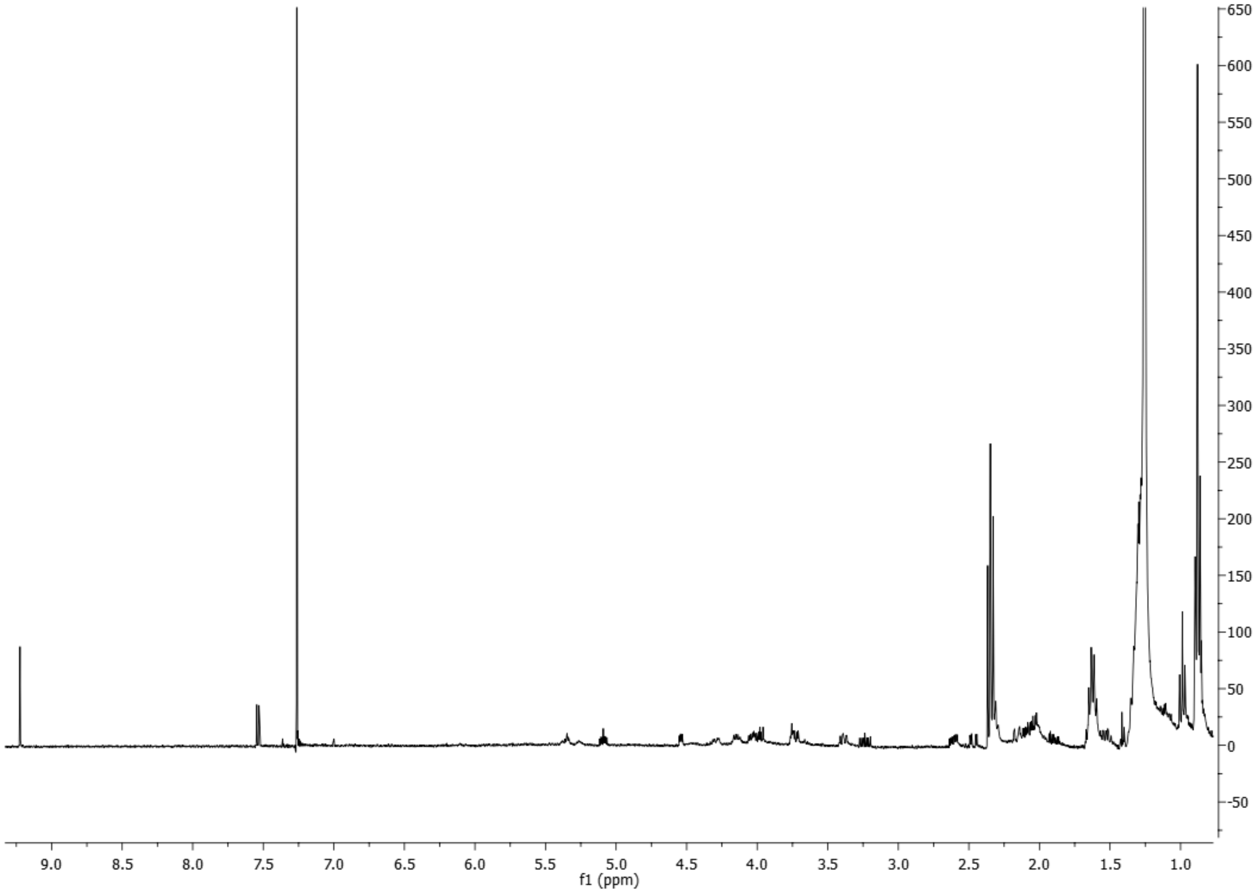

Figure S9. ^{13}C NMR spectrum of compound **2** ($CDCl_3$).

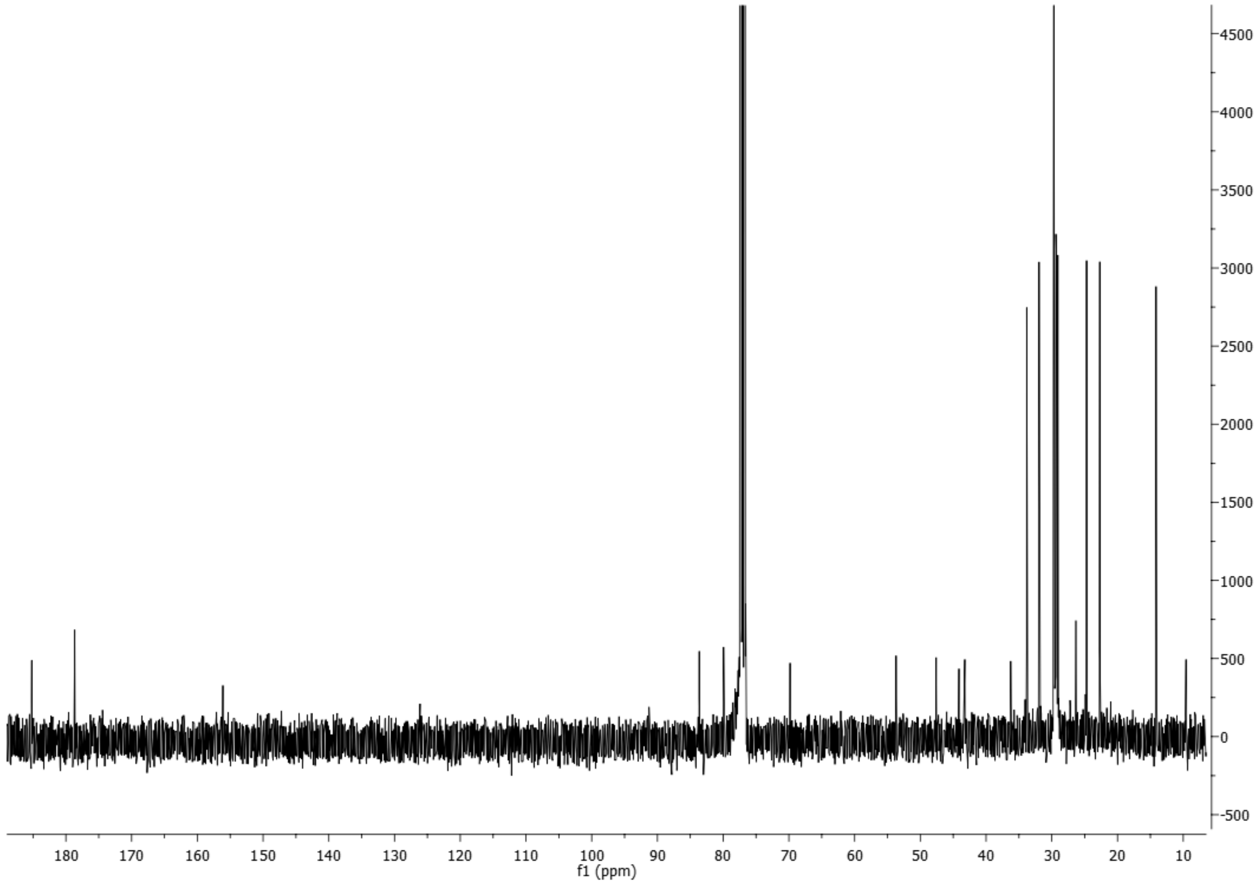

Figure S10. HSQC spectrum of compound **2** ($CDCl_3$).

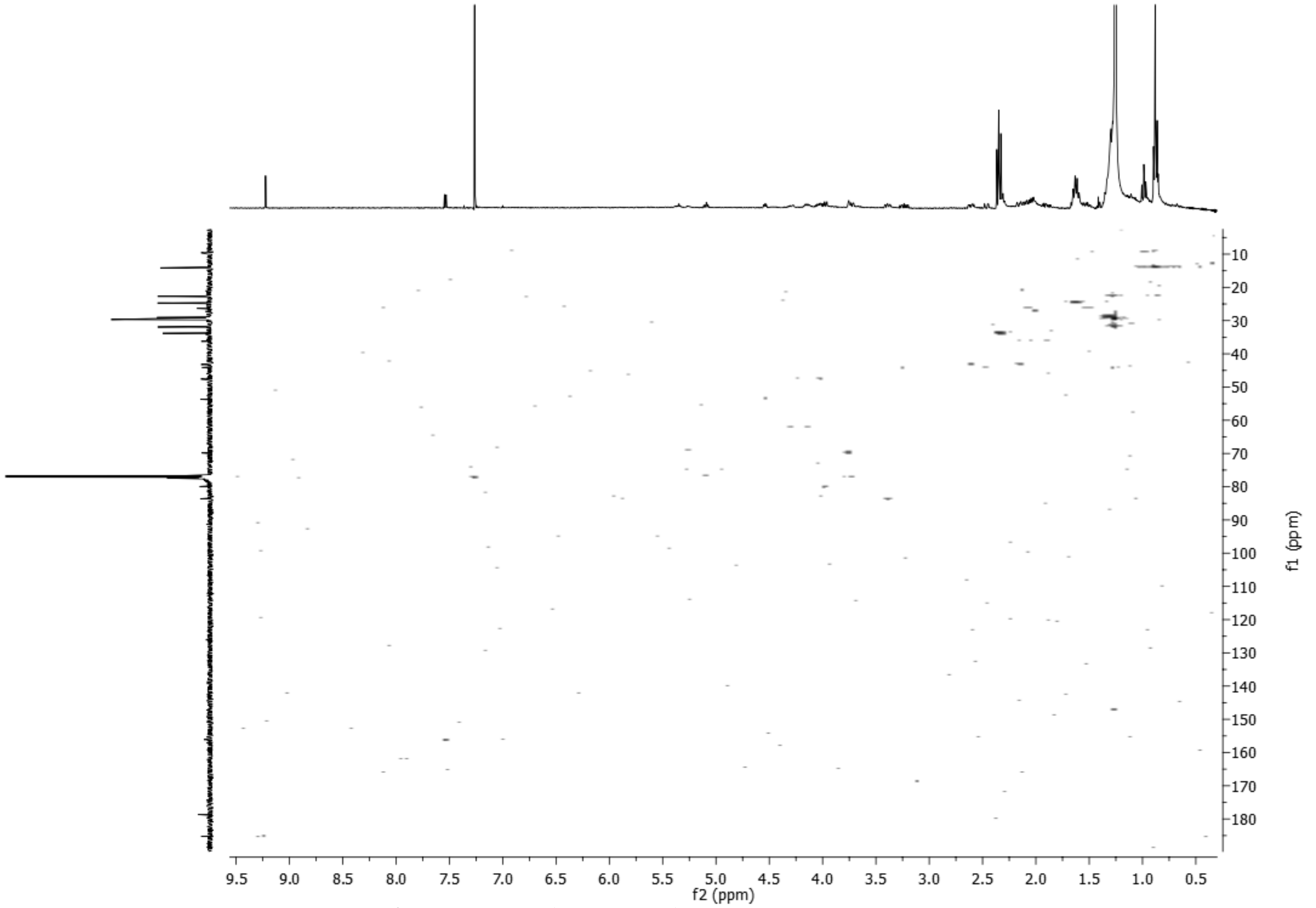

Figure S11. HMBC spectrum of compound **2** ($CDCl_3$).

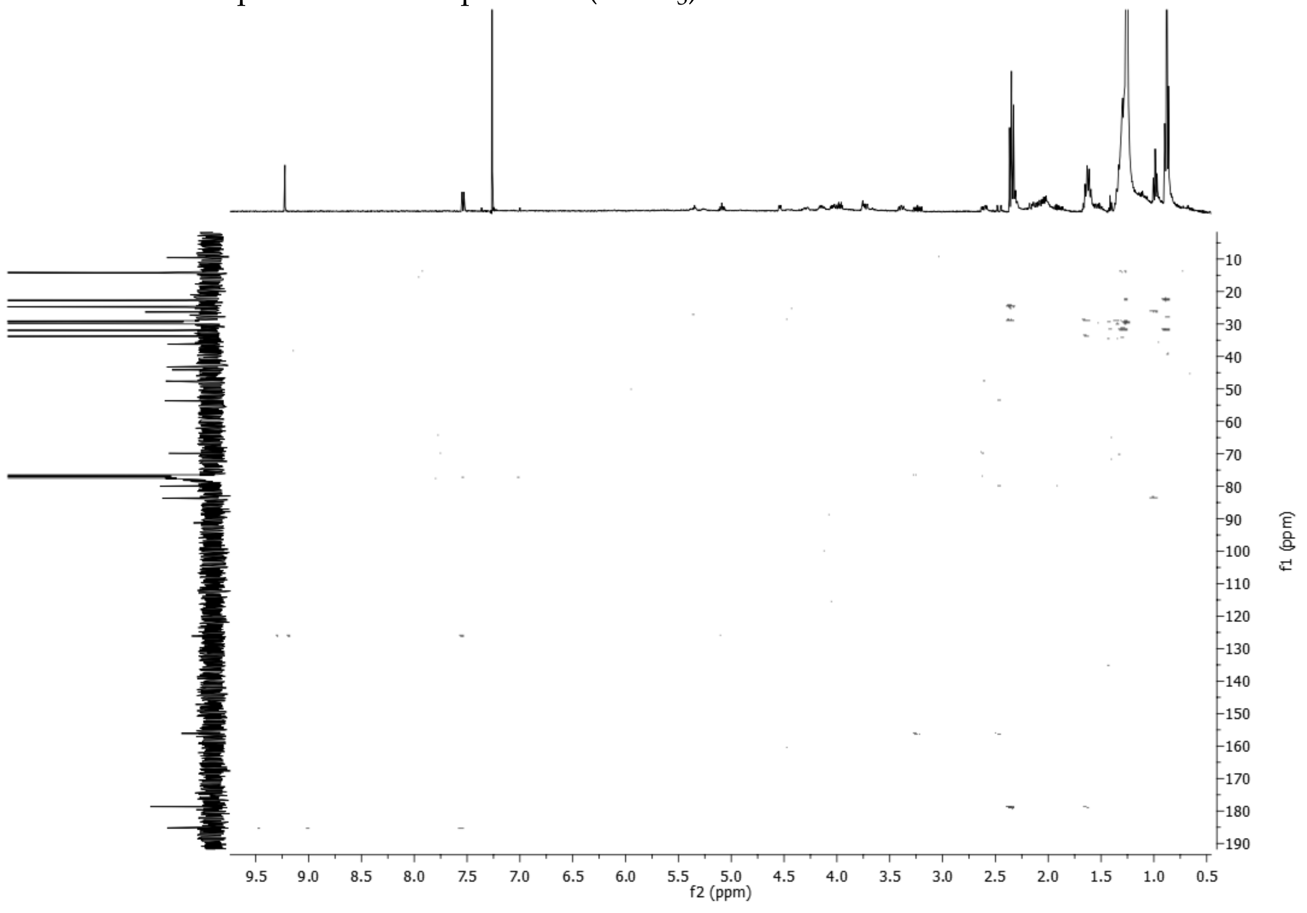

Figure S12. COSY spectrum of compound **2** ($CDCl_3$).

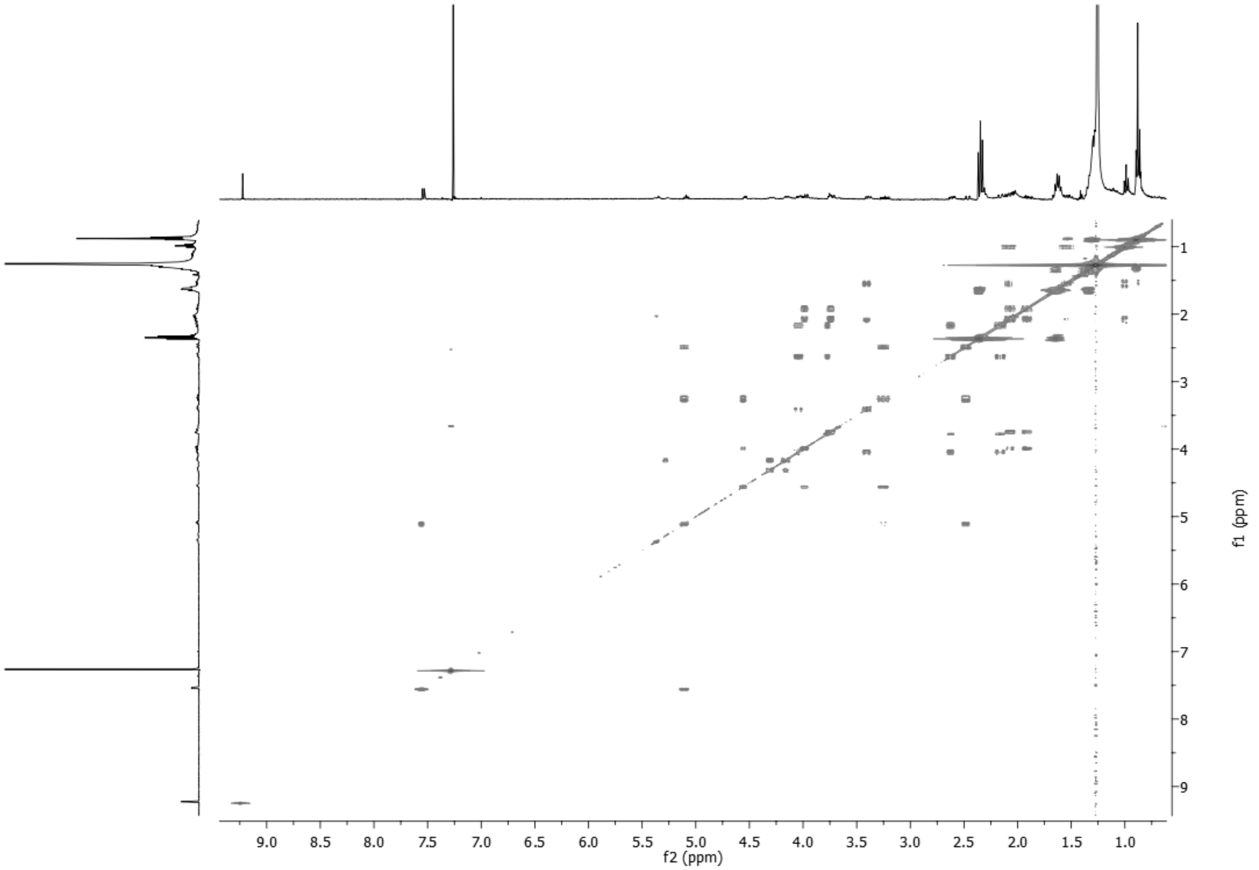

Figure S13. HRMS spectrum of compound **2** (Zoom) in negative ionization.

Elemental Composition Report

Page 1

Single Mass Analysis

Tolerance = 5.0 PPM / DBE: min = -1.5, max = 50.0
Element prediction: Off
Number of isotope peaks used for i-FIT = 3

Monoisotopic Mass, Even Electron Ions
2012 formula(e) evaluated with 20 results within limits (up to 50 best isotopic matches for each mass)
Elements Used:
C: 0-95 H: 0-182 O: 0-40 Na: 0-1 Cl: 0-3 Br: 0-8

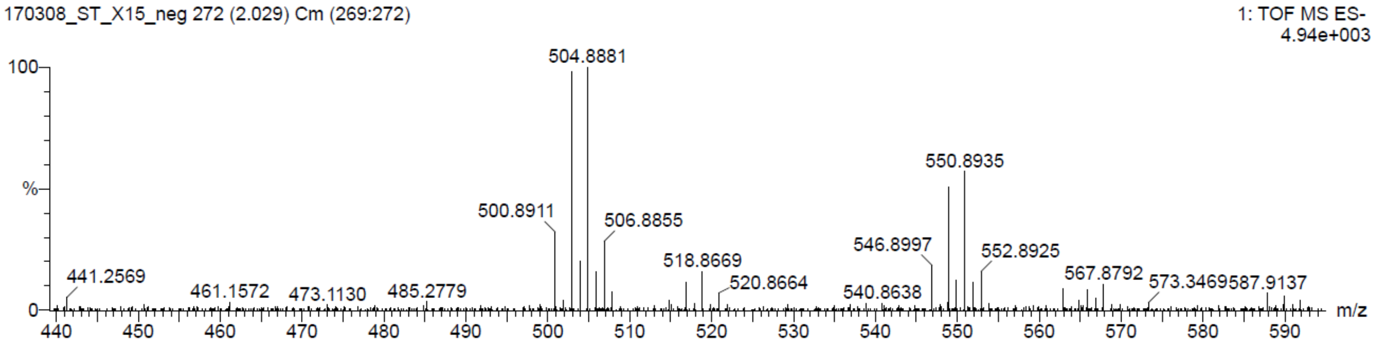

Minimum:				-1.5				
Maximum:		5.0	5.0	50.0				
**Mass**	**Calc. Mass**	**mDa**	**PPM**	**DBE**	**i-FIT**	**Norm**	**Conf(%)**	**Formula**
500.8911	500.8888	2.3	4.6	1.5	137.1	0.131	87.75	C13 H21 O4 Na Br3
	500.8912	-0.1	-0.2	4.5	139.1	2.147	11.68	C15 H20 O4 Br3

Figure S14. ^{1}H NMR spectrum of compound **3** (acetone-d_6).

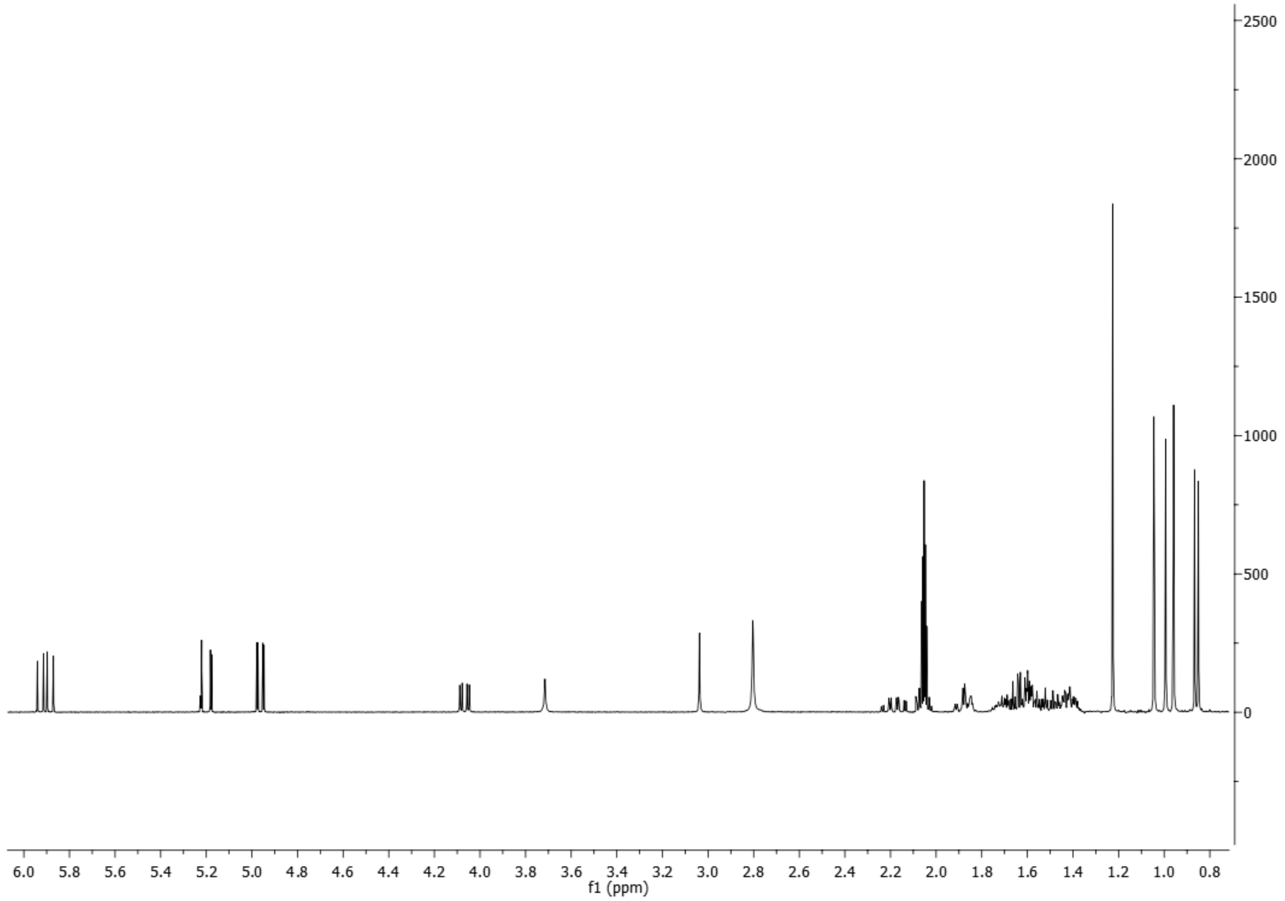

Figure S15. ^{13}C NMR spectrum of compound **3** (acetone-d_6).

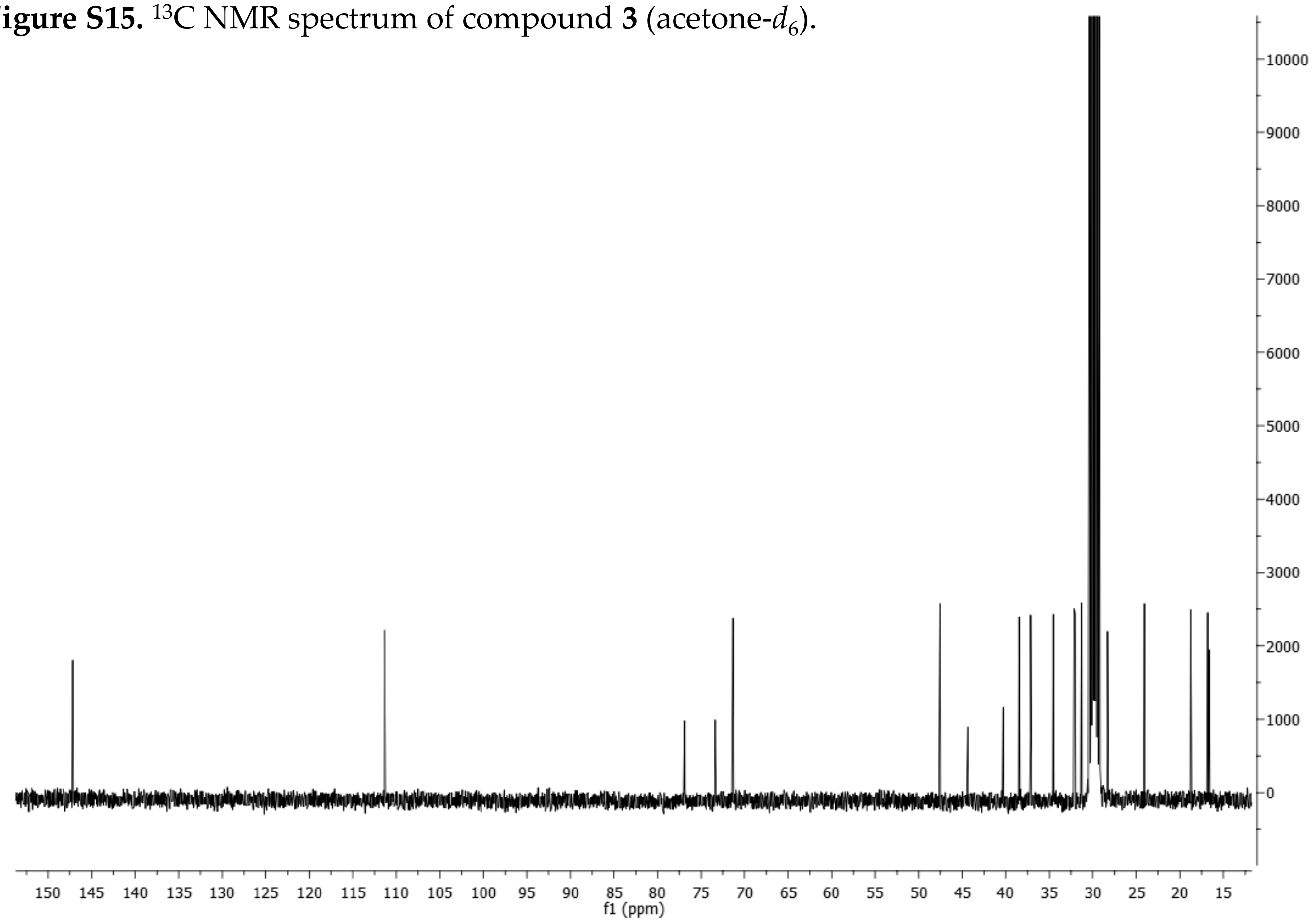

Figure S16. HSQC spectrum of compound **3** (acetone-d_6).

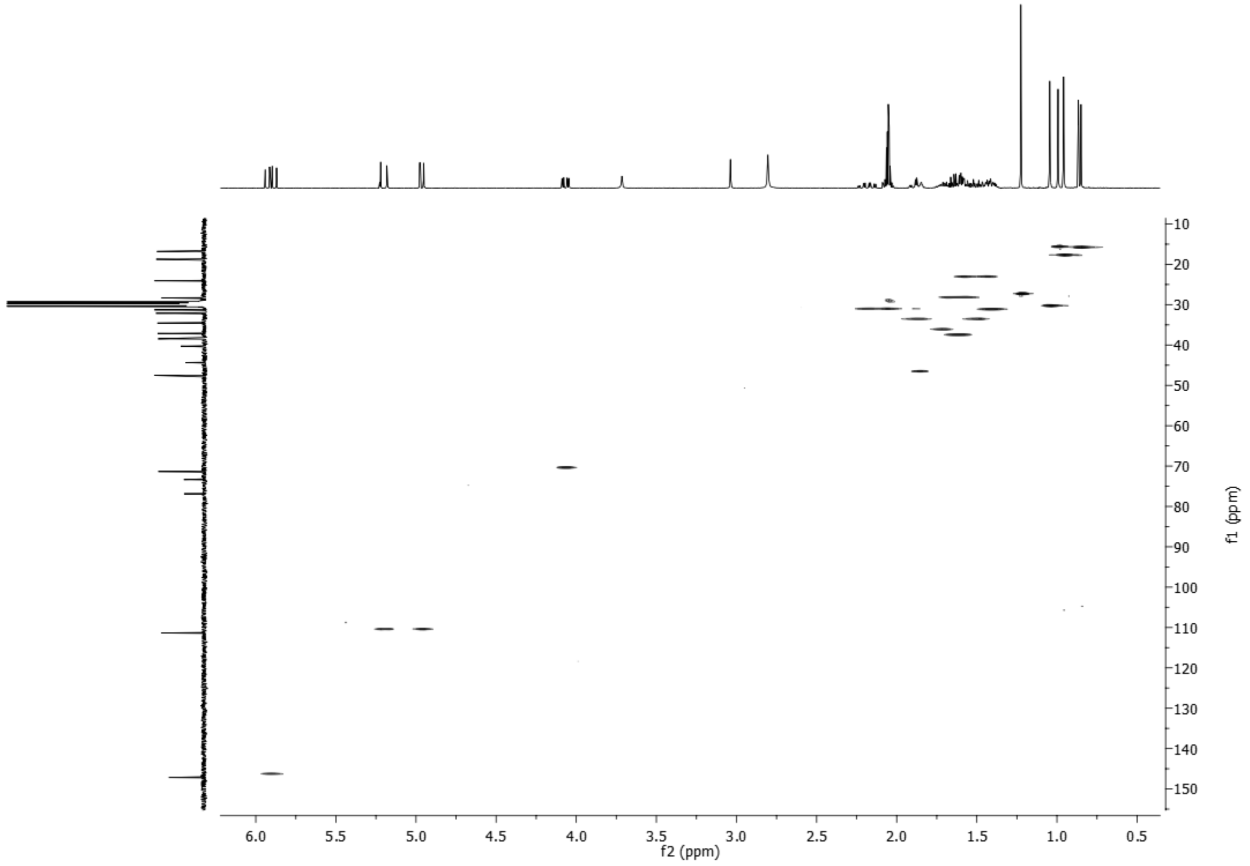

Figure S17. HMBC spectrum of compound **3** (acetone-d_6).

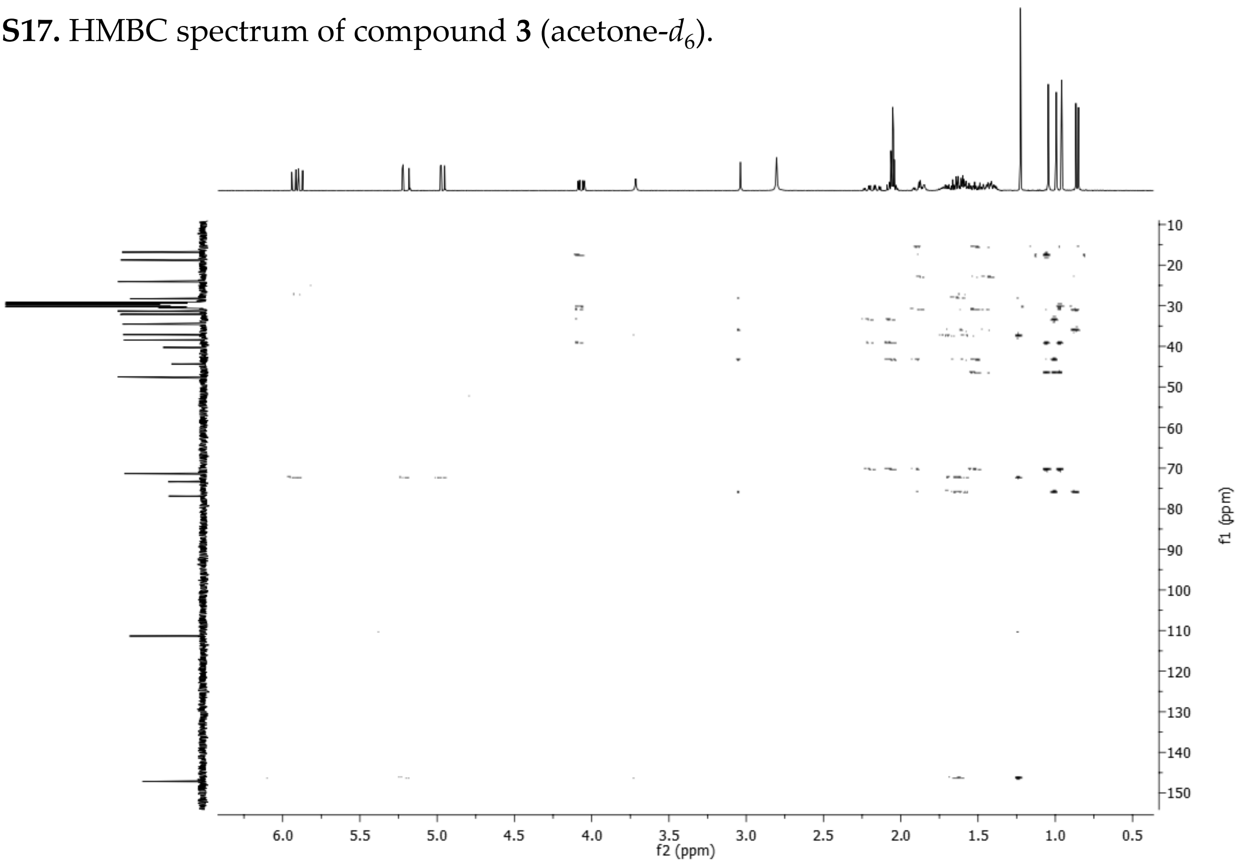

Figure S18. COSY spectrum of compound **3** (acetone-d_6).

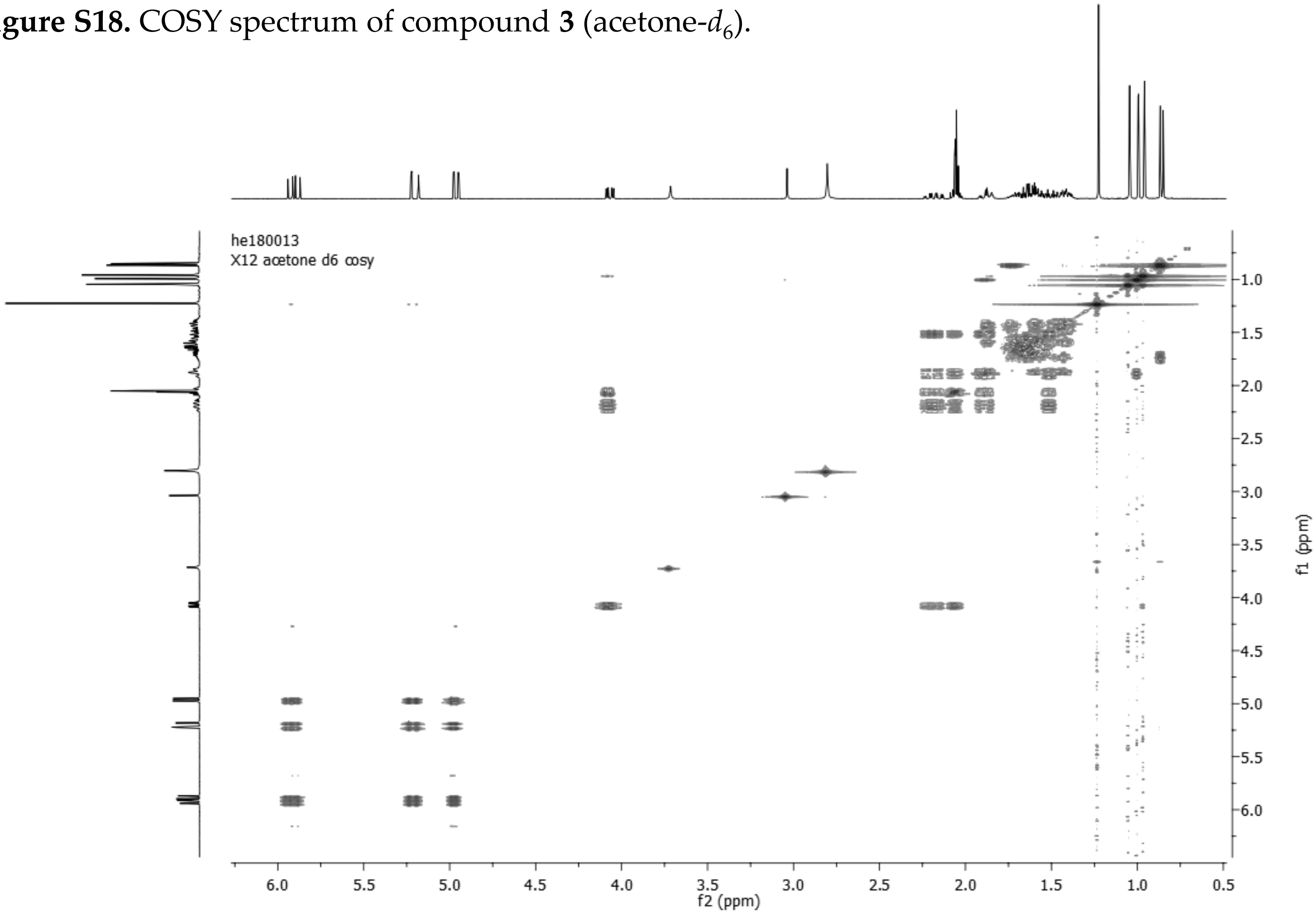

Figure S19. NOESY spectrum of compound **3** (acetone-d_6).

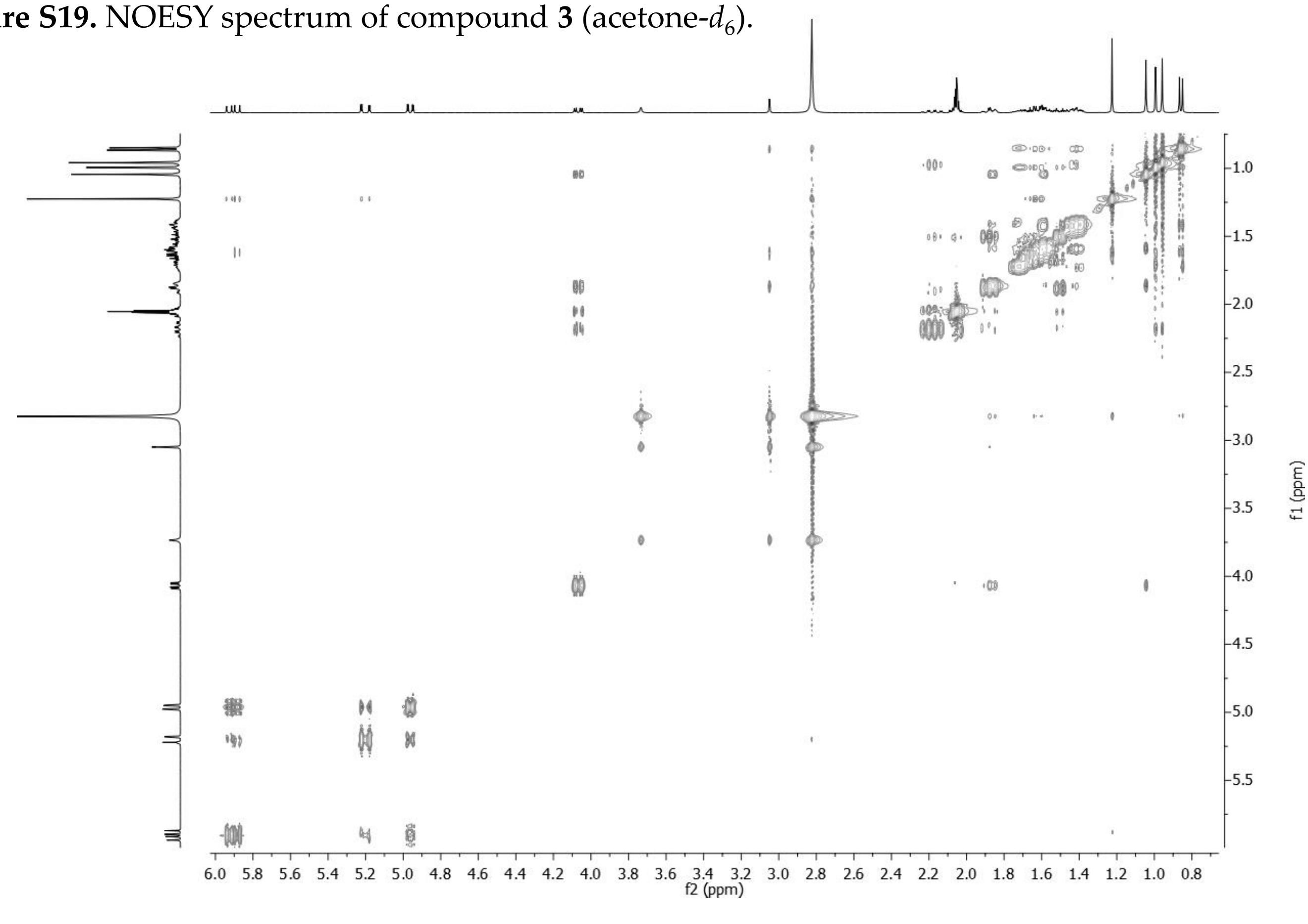

Figure S20. HRMS spectrum of compound **3** (Zoom) in positive ionization.

Elemental Composition Report

Single Mass Analysis

Tolerance = 5.0 PPM / DBE: min = -1.5, max = 50.0
Element prediction: Off
Number of isotope peaks used for i-FIT = 3

Monoisotopic Mass, Even Electron Ions
767 formula(e) evaluated with 1 results within limits (up to 50 best isotopic matches for each mass)
Elements Used:
C: 0-95 H: 0-182 O: 0-40 Na: 0-1 Cl: 0-3 Br: 0-8

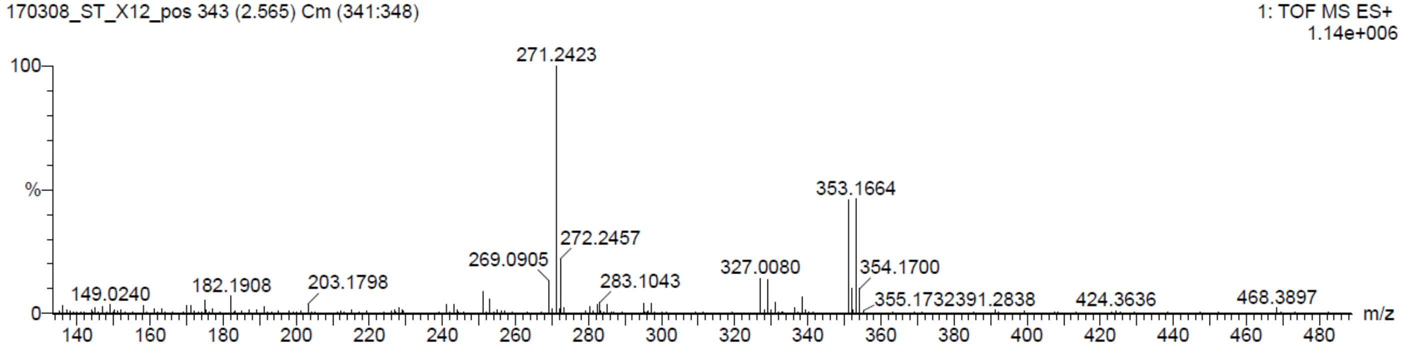

	Mass	Calc. Mass	mDa	PPM	DBE	i-FIT	Norm	Conf(%)	Formula
Minimum:					-1.5				
Maximum:			5.0	5.0	50.0				
	351.1681	351.1687	-0.6	-1.7	4.5	629.3	n/a	n/a	C20 H32 Br

Figure S21. ^{1}H NMR spectrum of compound **4** (acetone-d_6).

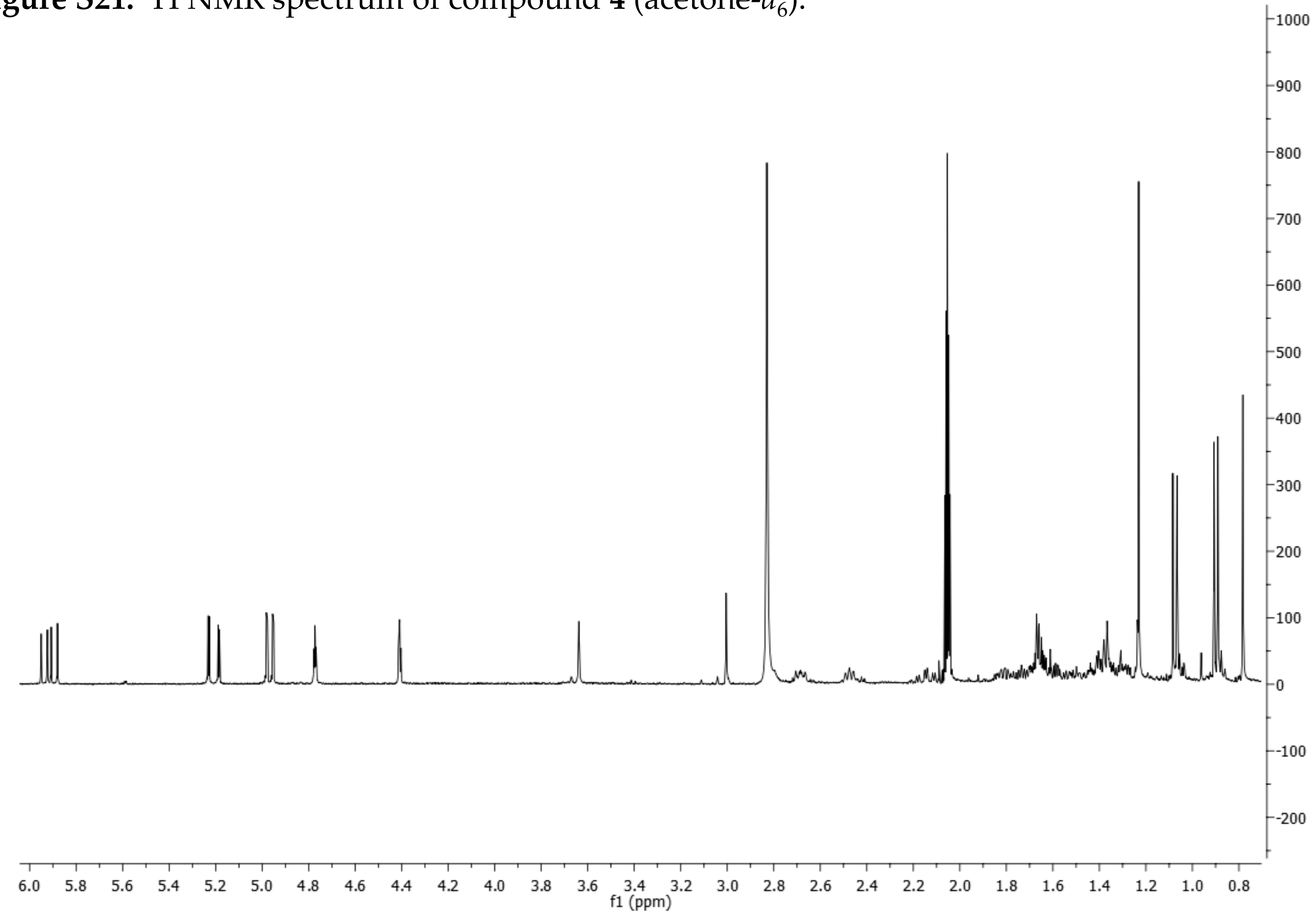

Figure S22. ^{13}C NMR spectrum of compound **4** (acetone-d_6).

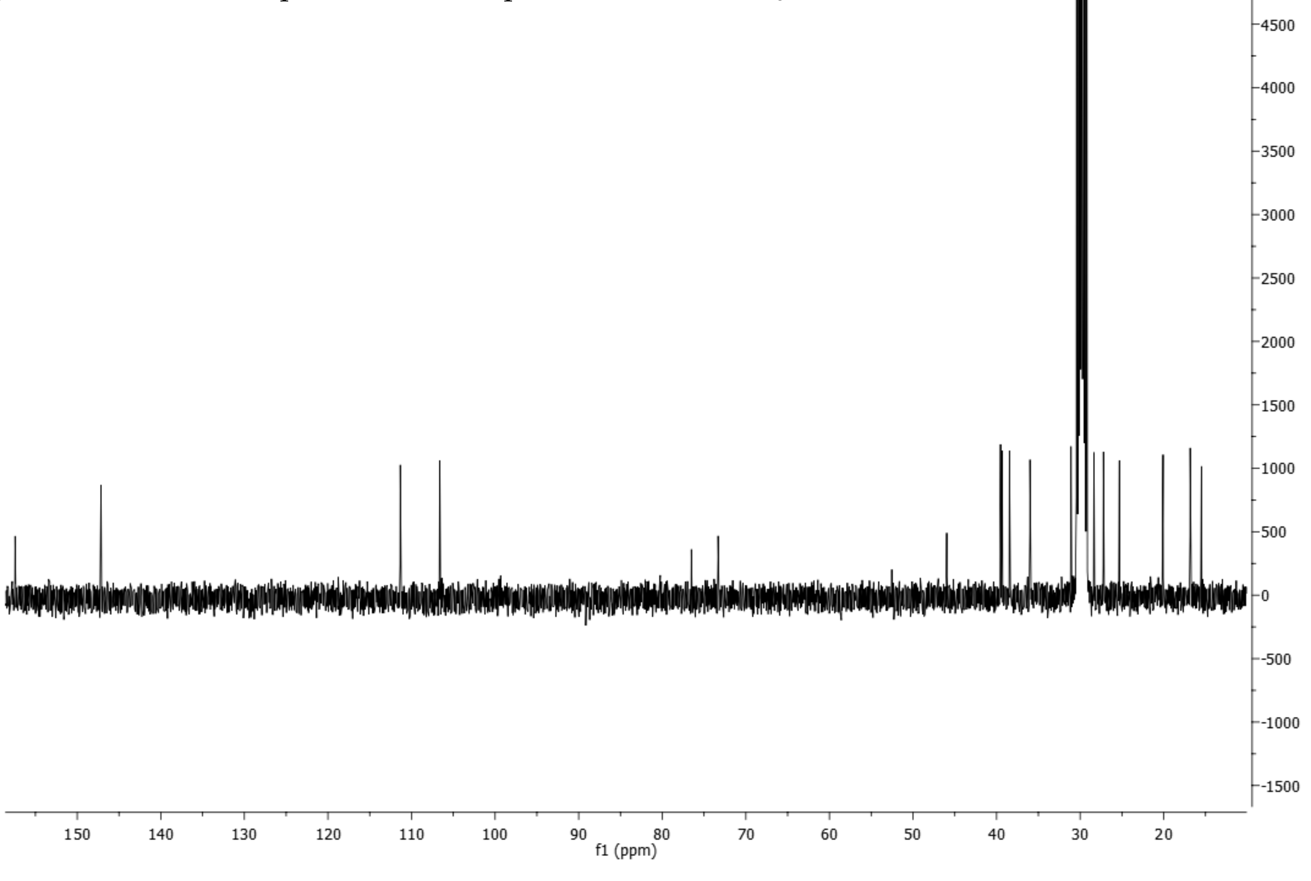

Figure S23. HSQC spectrum of compound **4** (acetone-d_6).

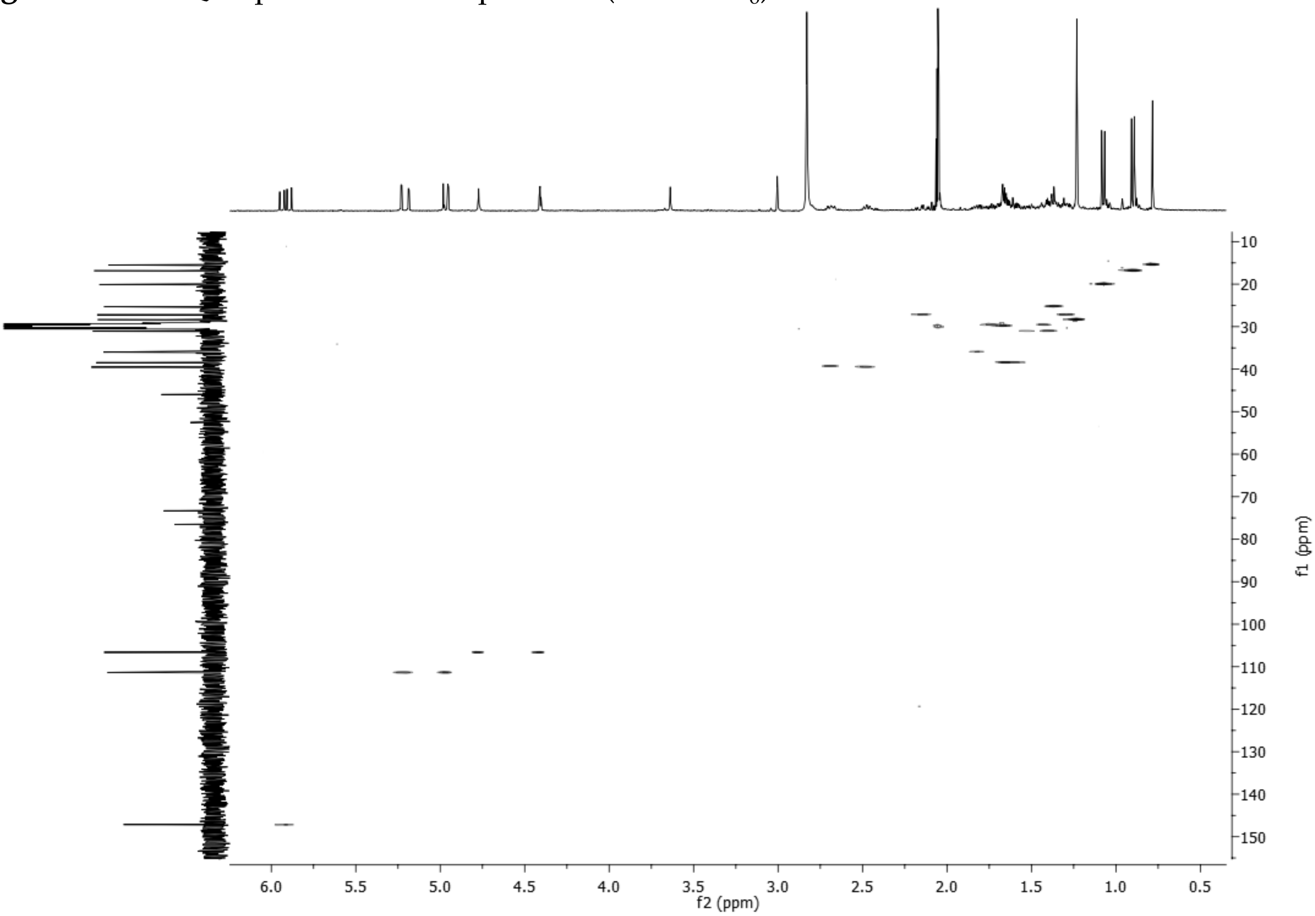

Figure S24. HMBC spectrum of compound **4** (acetone-d_6).

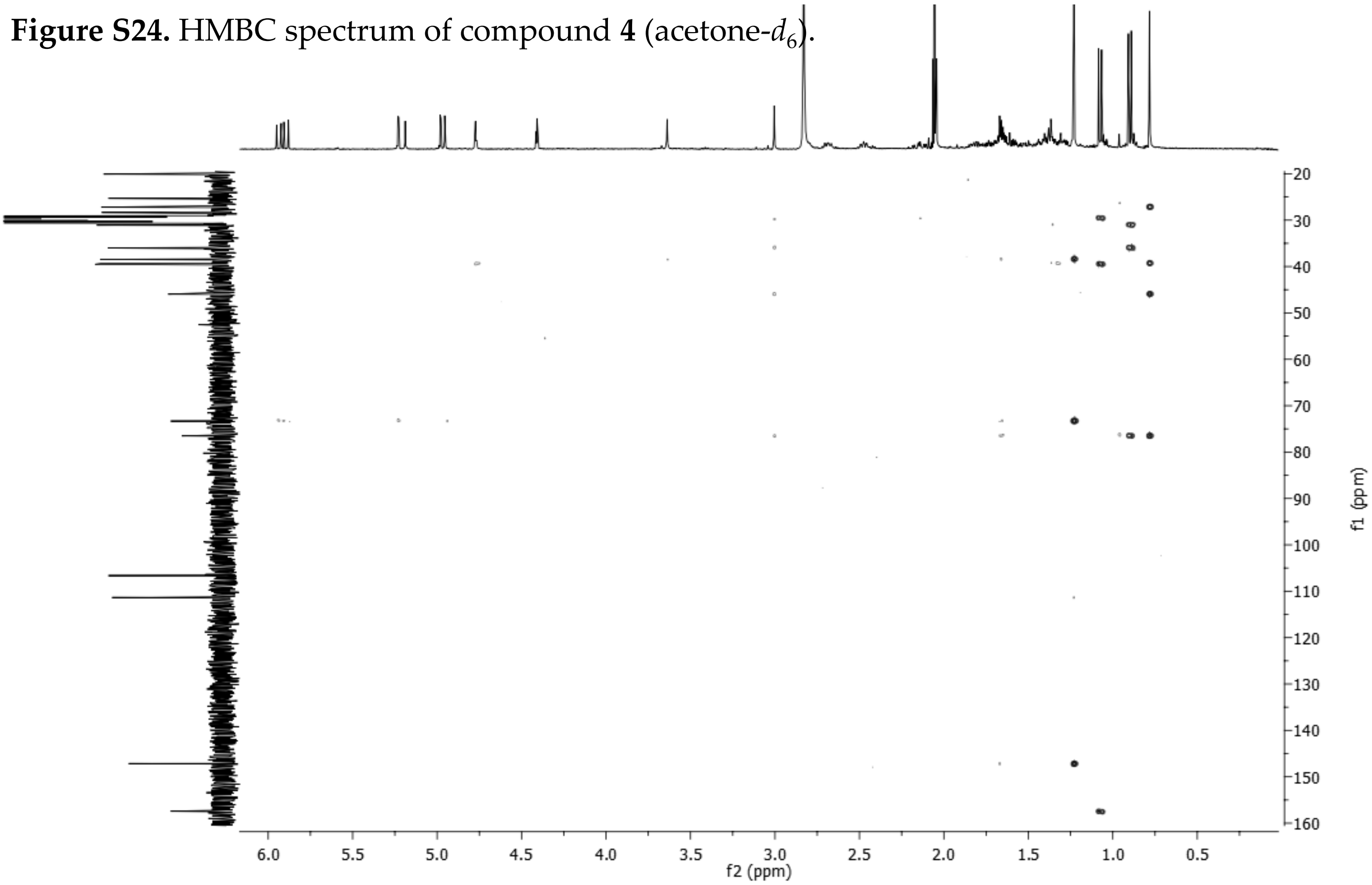

Figure S25. COSY spectrum of compound **4** (acetone-d_6).

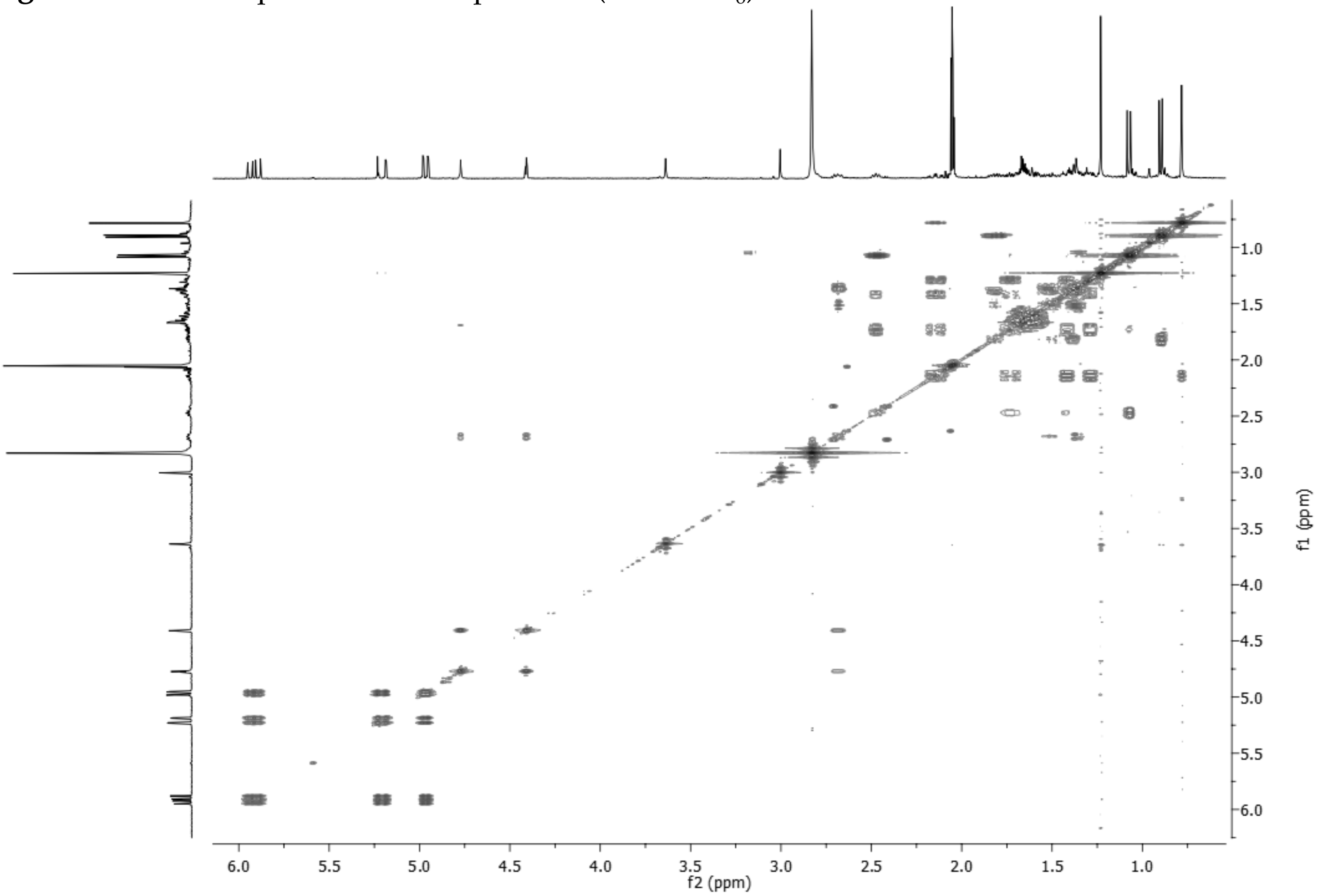

Figure S26. NOESY spectrum of compound **4** (acetone-d_6).

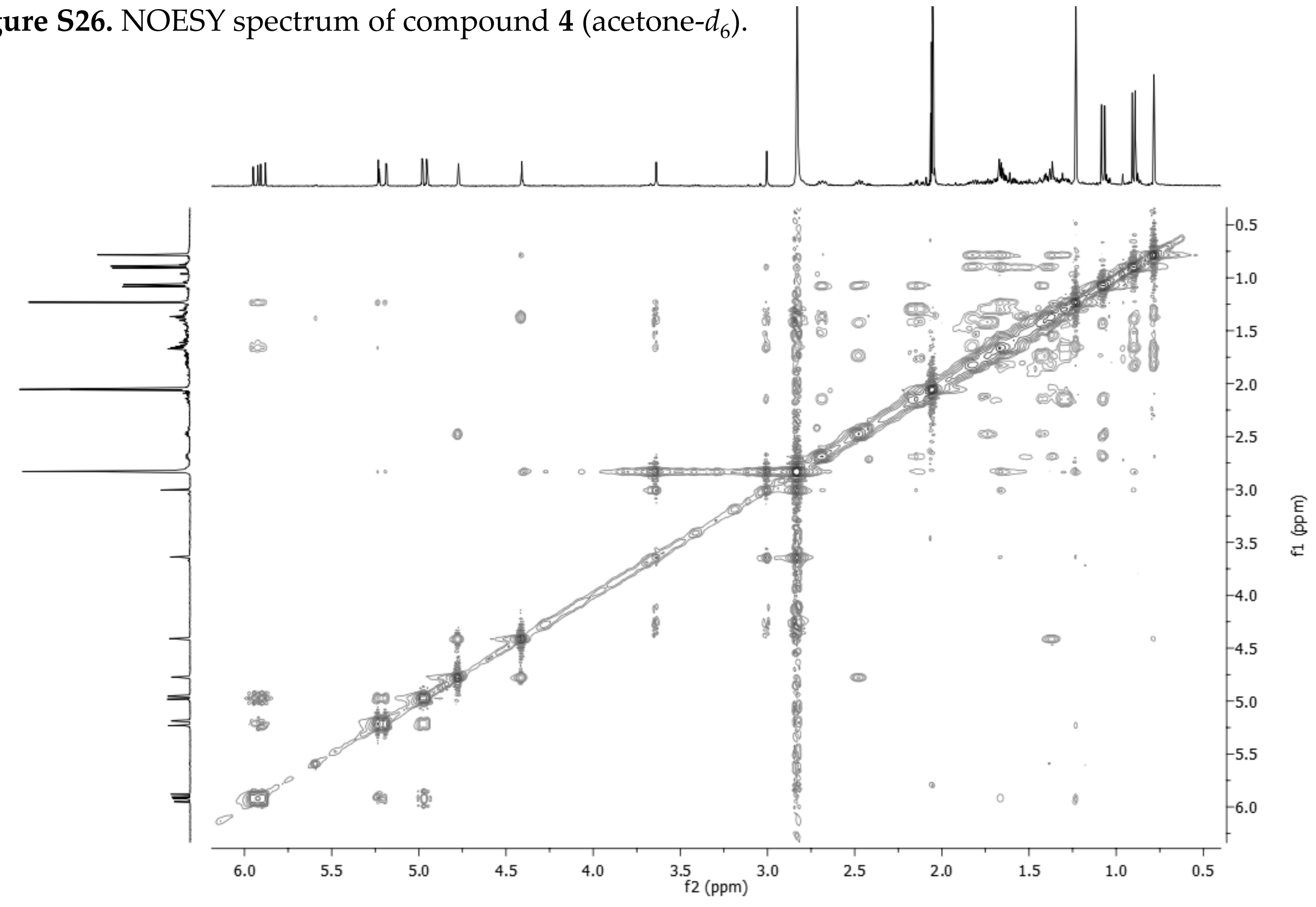

Figure S27. HRMS spectrum of compound **4** (Zoom) in negative ionization.

Elemental Composition Report

Page 1

Single Mass Analysis

Tolerance = 5.0 PPM / DBE: min = -1.5, max = 50.0
Element prediction: Off
Number of isotope peaks used for i-FIT = 3

Monoisotopic Mass, Even Electron Ions
769 formula(e) evaluated with 1 results within limits (up to 50 best isotopic matches for each mass)
Elements Used:
C: 0-95 H: 0-182 O: 0-40 Na: 0-1 Cl: 0-3 Br: 0-8

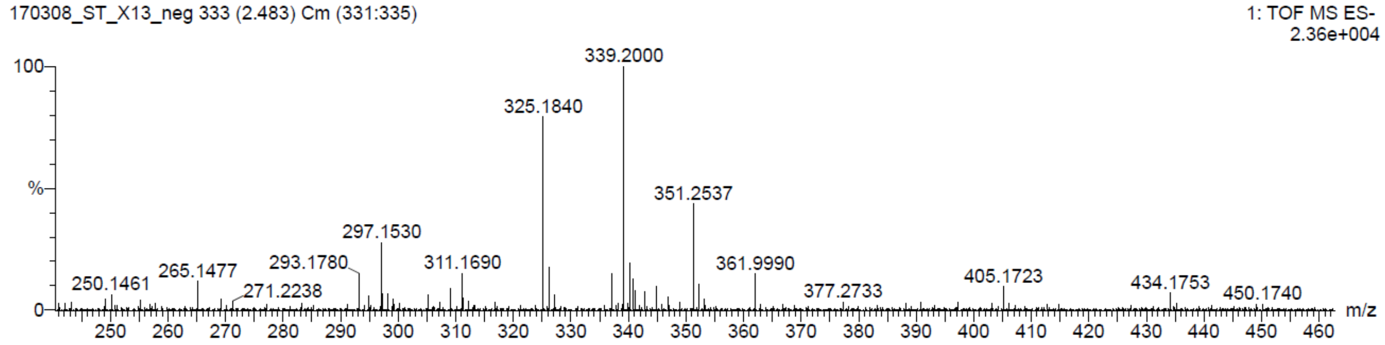

Minimum:					-1.5				
Maximum:			5.0	5.0	50.0				
	Mass	Calc. Mass	mDa	PPM	DBE	i-FIT	Norm	Conf(%)	Formula
	351.2537	351.2535	0.2	0.6	4.5	207.0	n/a	n/a	C21 H35 O4

Figure S28. HRMS spectrum of compound **4** (Zoom) in positive ionization.

Elemental Composition Report

Page 1

Single Mass Analysis

Tolerance = 5.0 PPM / DBE: min = -1.5, max = 50.0
Element prediction: Off
Number of isotope peaks used for i-FIT = 3

Monoisotopic Mass, Even Electron Ions
387 formula(e) evaluated with 1 results within limits (up to 50 best isotopic matches for each mass)
Elements Used:
C: 0-95 H: 0-182 O: 0-40 Na: 0-1 Cl: 0-3 Br: 0-8

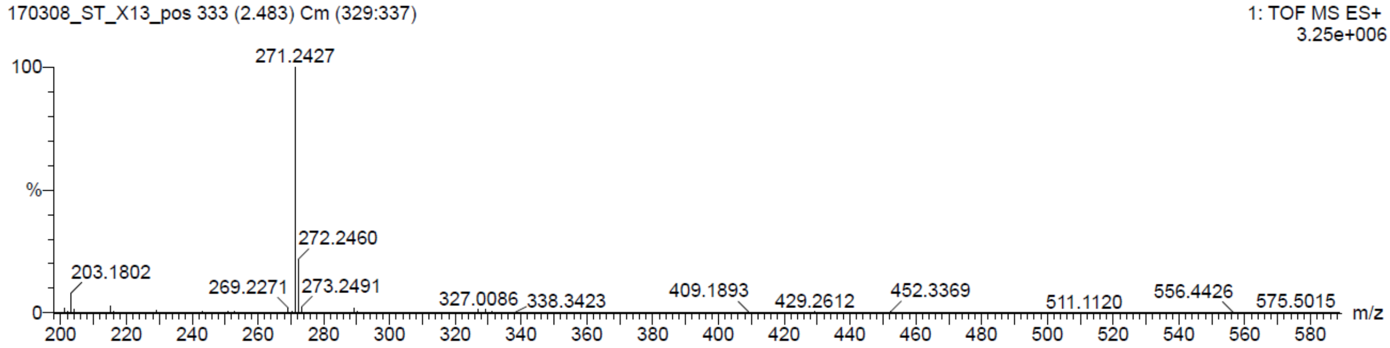

Minimum:					-1.5				
Maximum:			5.0	5.0	50.0				
	Mass	Calc. Mass	mDa	PPM	DBE	i-FIT	Norm	Conf(%)	Formula
	271.2427	271.2426	0.1	0.4	5.5	709.3	n/a	n/a	C20 H31

Figure S29. Structures of known compounds identified in *L. obtusa* extract.

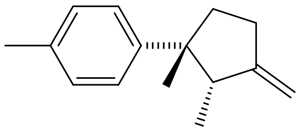

laurene (**6**)

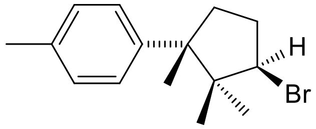

α-bromocuparene (**7**)

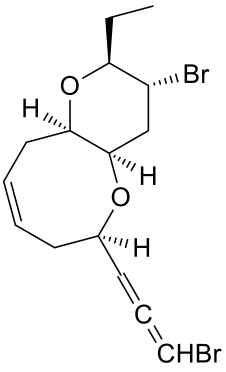

microcladallene A (**8**)

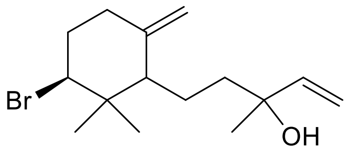

β-snyderol (**9**)
